# Spatiotemporal distribution of the glycoprotein pherophorin II reveals stochastic geometry of the growing ECM of *Volvox carteri*

**DOI:** 10.1073/pnas.2425759122

**Published:** 2025-08-12

**Authors:** Benjamin von der Heyde, Anand Srinivasan, Sumit Kumar Birwa, Eva Laura von der Heyde, Steph S. M. H. Höhn, Raymond E. Goldstein, Armin Hallmann

**Affiliations:** ^a^Department of Cellular and Developmental Biology of Plants, University of Bielefeld, Bielefeld 33615, Germany; ^b^Department of Applied Mathematics and Theoretical Physics, Centre for Mathematical Sciences, University of Cambridge, Cambridge CB3 0WA, United Kingdom

**Keywords:** extracellular matrix, multicellularity, *Volvox*, self-assembly

## Abstract

The evolution of multicellularity involved the transformation of a simple cell wall of unicellular ancestors into a complex, multifunctional extracellular matrix (ECM). A suitable model organism to study the formation and expansion of an ECM during ontogenesis is the multicellular green alga *Volvox carteri*, which, along with the related volvocine algae, produces a complex, self-organized ECM composed of multiple substructures. These self-assembled structures primarily consist of hydroxyproline-rich glycoproteins, a major component of which is pherophorins. To investigate the geometry of the growing ECM, we fused the *yfp* gene with the gene for pherophorin II (PhII) in *V. carteri*. Confocal microscopy reveals PhII:YFP localization at key ECM structures, including the boundaries of compartments surrounding each somatic cell and the outer surface of the organism. Image analysis during the life cycle allows the stochastic geometry of growing compartments to be quantified; their areas and aspect ratios exhibit robust gamma distributions and exhibit a structural transition from a tight polygonal to a looser acircular packing geometry with stable eccentricity over time, evoking parallels and distinctions with the behavior of hydrated foams. These results provide quantitative insight into a general, open question in biology: how do cells collectively produce a complex structure external to themselves in a robust and accurate manner?

## Significance

The extracellular matrix (ECM) plays important structural, developmental, and physiologic roles in animals, fungi, plants, and algae and was particularly important in evolutionary transitions from unicellular to multicellular organisms. As the ECM is by definition external to cells, there must necessarily be an aspect of self-assembly involved in its generation, yet little is known regarding such processes even in the simplest multicellular species. Here, we report the development of a transgenic strain of the multicellular green alga *Volvox carteri*, in which a key ECM protein, pherophorin II, is fused with yellow fluorescent protein, allowing a quantitative study of stochastic ECM geometry and growth dynamics in this system, revealing behavior reminiscent of, yet distinct from, the hydration of foams.

Throughout the history of life, one of the most significant evolutionary transitions was the formation of multicellular eukaryotes. In most lineages that evolved multicellularity, including animals, fungi, and plants, the extracellular matrix (ECM) has been a key mediator of this transition by connecting, positioning, and shielding cells (1–4). The same holds for multicellular algae such as *Volvox carteri* (Chlorophyta) and its multicellular relatives within volvocine green algae which developed a remarkable array of advanced traits in a comparatively short amount of evolutionary time—oogamy, asymmetric cell division, germ-soma division of labor, embryonic morphogenesis and a complex ECM (5–8)—rendering them uniquely suited model systems for examining evolution from a unicellular progenitor to multicellular organisms with different cell types (5, 6, 8–10). In particular, *V. carteri*’s distinct, multilayered ECM makes it a model organism for investigating mechanisms underlying ECM growth and the dynamics of its structures alongside the positions of the cells that secrete its components. Building on recent protocols for stable expression of fluorescently labeled proteins in *V. carteri* (11–13), we present here a transgenic strain revealing localization of the glycoprotein pherophorin II and the first in vivo study of the stochastic geometry of a growing ECM.

**Fig. 1.**
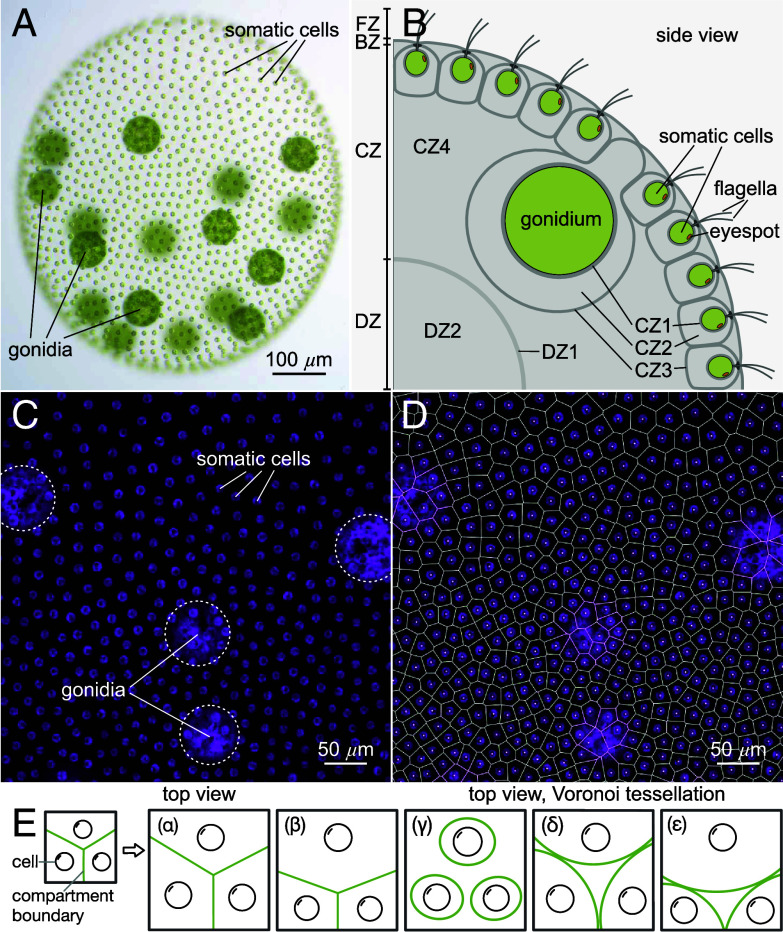
Phenotype, ECM architecture, and cell distribution of *V. carteri*. (*A*) Adult female (wild-type). Somatic cells each bear an eyespot and two eukaryotic flagella (motile cilia), while deeper-lying gonidia are flagella-less. All cells have a single large chlorophyll-containing chloroplast (green). (*B*) Zones of the ECM, as described in text. (*C*) Image utilizing autofluorescence of the chlorophyll (magenta) to define cell positions. (*D*) Semiautomated readout of somatic cell positions in (*C*) followed by a Voronoi tessellation gives an estimate of the ECM neighborhoods of somatic cells. (*E*) Hypothetical expansion dynamics (α-ε) of a compartmentalized ECM, e.g., the *Volvox* CZ2/3 (panel *B*). Factors include wall merging & adhesion (exemplified by α,δ), permeability (γ), and cell–cell heterogeneous ECM production (β,ε).

*V. carteri* usually reproduces asexually (Fig. 1*A*). Sexual development is triggered by exposure to heat or a species-specific glycoprotein sex inducer, which results in development of sperm-packet-bearing males and egg-bearing females (14–17). In the usual asexual development, *V. carteri* consists of ∼2,000 biflagellated somatic cells resembling *Chlamydomonas* in their morphology, arranged in a monolayer at the surface of a sphere, and ∼16 much larger, nonmotile, asexual reproductive cells (gonidia) that constitute the germline, lying just below the somatic cell layer (5–7, 18, 19). The somatic cells are specialized for photoreception and motility, and perform ECM biosynthesis jointly with the gonidia; for phototaxis, they must be positioned correctly within the ECM at the surface of the organism (14, 20, 21).

The ECM of *V. carteri* has been studied in the past decades from the perspective of structure and composition, developmental, mechanical properties, cellular interactions, molecular biology and genetics, and evolution (11, 14, 20–22). In the ontogenesis of *V. carteri*, ECM biosynthesis in juveniles begins while inside their mothers and only after all cell divisions and the process of embryonic inversion are completed. Cells of the juveniles then continuously excrete large quantities of ECM building blocks which are integrated into their ECM, producing an enormous increase in the size; within 48 h the volume increases ∼3,000-fold as the diameter increases from ∼70μm to ∼1mm, raising the question of how ECM geometry and relative cell positions transform during growth. In the adult, the ECM accounts for ∼99% of the organism’s volume and consists of morphologically distinct structures with a defined spatial arrangement. Based on electron microscopy, a nomenclature was established (20) that defines four main ECM zones: flagellar zone (FZ), boundary zone (BZ), cellular zone (CZ), and deep zone (DZ), which are further subdivided (Fig. 1*B*). The CZ3 forms the ECM compartment boundaries of individual cells and the BZ constitutes the outer surface of the organism. CZ3 and BZ show a higher electron density than the ECM within the compartments or the ECM in the interior below the cell layer (CZ4 and DZ2) (6, 7, 20). CZ3 and BZ therefore appear to consist of a firmer, more robust ECM, while CZ4 and DZ2 appear to be more gelatinous.

The ECM of volvocine algae predominantly consists of hydroxyproline-rich glycoproteins (HRGPs), which are also a major component of the ECM of embryophytic land plants (14, 21, 23–25), but contains no cellulose. In *V. carteri* and other volvocine algae, a large family of HRGPs, the pherophorins, are expected to constitute the main building material of the ECM (17, 22, 26–29). Just in *V. carteri*, 118 members of the pherophorin family were discovered in the genome (11, 30); pherophorin genes are typically expressed in a cell type–specific manner (31) that is constitutive or induced either by the sex inducer or wounding (17, 22, 26, 28, 29, 32). The dumbbell-like pherophorin structure has two globular domains separated by a rod-shaped, highly proline-rich one that varies considerably in length (14, 21, 28, 29). These prolines undergo posttranslational modification to hydroxyprolines; pherophorins are also strongly glycosylated (14, 21, 22, 33, 34). For two pherophorins the polymerization into an insoluble fibrous network was shown in vitro (26); some have already been localized in the ECM or in ECM fractions (11, 13, 22, 27, 29).

To investigate the ECM, we determined that pherophorin II (PhII) was the appropriate one to label fluorescently, as it is firmly integrated into the ECM. It is a 70 kDa glycoprotein with constant, weak background expression but also strong and immediate inducibility by the sex inducer (17, 35, 36). Because it can only be extracted under harsh conditions, it is thought to be a component of the insoluble part of the somatic CZ (17, 35, 36). We fused one of the nine (28, 30) gene copies of PhII with the *yfp* gene. The corresponding DNA construct was stably integrated into the genome by particle bombardment. The expression of fluorescent PhII:YFP was confirmed through confocal laser scanning microscopy (CLSM); it was found in the boundary zone and compartment boundaries of the cellular zones.

We suggest that a detailed study of the stochastic geometry of a growing ECM made possible by this strain of *Volvox* will provide insight into a more general question in biology: how do cells robustly produce structures external to themselves? There is no physical picture of how the intricate geometry of the *Volvox* ECM arises through what must be a self-organized process of polymer crosslinking (37) and hydration. While information on a structure’s growth dynamics can be inferred from its evolving shape, as done for animal epithelial cells (38), this connection has not been made for *Volvox*. The dynamics of the compartmental geometry (Fig. 1*E*) give clues as to wall material properties (including permeability and adhesion) and by extension the “autonomy” (degree of spatial constraint) of ECM deposition during growth. Some hypothetical regimes [Fig. 1*E* (β and ε)] provide readouts of cell-to-cell fluctuations in ECM production and expansion of the local region, making the PhII:YFP-stained CZ3 a valuable tool through which to understand spheroid morphogenesis. Some evoke visual analogies to two-dimensional dry [Fig. 1*E* (α−β)] and wet [Fig. 1*E* (γ−ε)] foams, observed for more than a century in tissues (39, 40), yet relatively unexplored in an ECM.

Recently (41), the somatic cells of *V. carteri* were located via their chlorophyll autofluorescence, from which the ECM neighborhoods were found by a surface Voronoi tessellation, as in Fig. 1 *C* and *D*. While the cells appear to be arranged in a quasi-regular pattern, their Voronoi areas exhibited a broad, skewed k-gamma distribution. Yet, the validity of the Voronoi model for actual ECM structures—such as the CZ3—during development has remained uncertain. And while such universal size and shape distributions have been identified in granular materials (42), mathematical constructs (43), epithelial tissues, and inert jammed matter (44), it is unknown whether this stochastic geometry also arises in a compartmentalized ECM. Subsequent work suggested that gamma-distributions may arise from bursty cellular ECM production (45). To investigate this and to answer the more fundamental questions raised above, we analyzed the geometry of the structure illuminated by PhII:YFP.

Our results show, first and foremost, that the CZ3 compartments remains a connected and nearly space-filling structure throughout development; its geometry provides a meaningful readout of the spheroid’s developmental dynamics during expansion. We find that this structure does not simply dilate during growth [globally 1E(α) or locally 1E(β)] but instead undergoes a foam-like structural transition [Fig. 1*E* (ε)] from an initial configuration that closely resembles Voronoi geometry. During this transition, we find that shape features (Table 1) exhibit the same stochastic geometry alluded to above: a cell-scale randomness in size and shape which strikingly coexists with robust organism-scale symmetries and anterior–posterior differentiation.

**Table 1.** Metric definitions

Metric	Symbol	Definition	Units
Somatic cell area	$a_{\mathrm{cell}}$	–	μm^2^
Somatic cell centroid	$x_{\mathrm{cell}}$	–	μm
CZ3 compartment area	$a_{\mathrm{cz}3}$	–	μm^2^
CZ3 compartment centroid	$x_{\mathrm{cz}3}$	–	μm
CZ3 compartment perimeter	$\ell_{\mathrm{cz}3}$	–	μm
CZ3 covariance matrix	Σ	Eq. **1**	μm^2^
Aspect ratio	α	$\sqrt{\lambda_{\max}/\lambda_{\min}}$	Unitless
Circularity	q	$\sqrt{4\pi a_{\mathrm{cz}3}}/\ell_{\mathrm{cz}3}$	Unitless
Somatic cell offset vector	Δx	$x_{\mathrm{cell}}-x_{\mathrm{cz}3}$	μm
Somatic cell offset	r	Δx	μm
Somatic cell offset (whitened)	r	$\sqrt{\Delta x\cdot \Sigma^{-1}\Delta x}$	Unitless
Voronoi error	$e_{V}$	vor ∩ cz3 / vor ∪ cz3	Unitless

## 1. Results

### 1.1. Vector Construction and Generation of Transformants Expressing PhII:YFP.

**Fig. 2.**
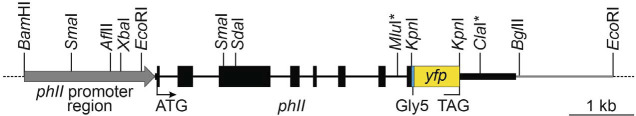
Schematic diagram of the transformation vector pPhII-YFP. The vector carries the complete *phII* gene including its promoter region. Exons (introns) are shown as black boxes (thin lines). Directly upstream of the TAG stop codon, a 0.7 kb fragment coding for a flexible penta-glycine spacer (cyan) and the codon-adapted coding sequence of *yfp* (mVenus) (yellow) were inserted in frame using an artificially introduced *Kpn*I site. The *Kpn*I was inserted before into the section between *Mlu*I and *Cla*I (asterisks). The vector backbone (dashed lines) comes from pUC18. For details, see *Materials and Methods*; the sequence of the vector insert is in *SI Appendix*, Fig. S3.

The pherophorin II gene (*phII*) was cloned from *V. carteri* genomic DNA including its promoter, 5^′^ and 3^′^ UTRs and all seven introns (Fig. 2). A sequence coding for a flexible penta-glycine spacer and the codon-adapted coding sequence of *yfp* were inserted directly upstream of the *phII* stop codon to produce a *phII-yfp* gene fusion. The obtained vector pPhII-YFP (Fig. 2) was sequenced and then used for stable nuclear transformation of the nitrate reductase-deficient *V. carteri* recipient strain TNit-1013 by particle bombardment. To allow for selection, the nonselectable vector pPhII-YFP was cotransformed with the selectable marker vector pVcNR15, which carries an intact *V. carteri* nitrate reductase gene and, thus, complements the nitrate reductase deficiency of strain TNit-1013. Screening for transformants was then achieved by using medium with nitrate as the nitrogen source. The transformants were investigated for stable genomic integration of the vector and, via confocal microscopy, for expression of the fluorescent protein at sufficient levels throughout their life cycle.

### 1.2. In Vivo Localization of Pherophorin II.

**Fig. 3.**
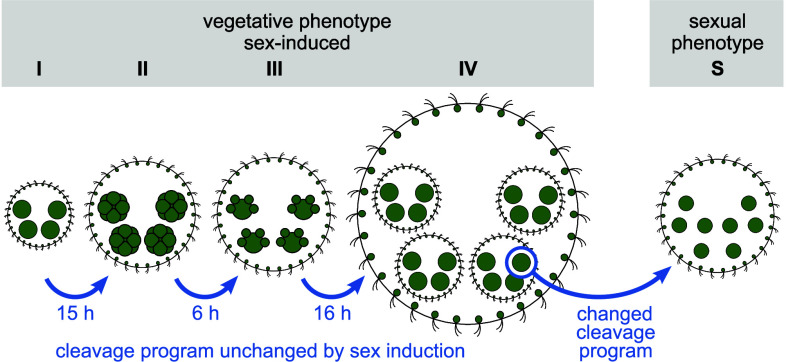
Stages of development of *V. carteri* females. Localization of PhII and quantification of ECM features were studied in 5 stages, four with vegetative (asexual) phenotype (I to IV) and one with the fully developed sexual phenotype (S). In stages I to IV, incubation with the sex inducer was short enough to increase PhII expression for adequate localization without changing the vegetative cleavage program.

As expected and detailed below, there are continuous changes in the amount of ECM expansion over the life cycle. While we primarily examine the parental somatic cell layer and surrounding ECM, the developmental stage of the next generation is used for a precise definition of five key stages used in the comparative analyses (Fig. 3). Stage I: freshly hatched young adults of equivalent circular radius R=106±6μm (*SI Appendix*, section 2.B), containing immature gonidia. Stage II [∼15 h post hatching (hph)]: middle-aged adults with R=221±22μm containing early embryos (4 to 8 cell stage). Stage III (∼21 hph): older middle-aged adults with R=244±15μm containing embryos before inversion. Stage IV (∼36 hph): old adults with R=422±6μm containing fully developed juveniles. Stage S: sexually developed adult females with R=265±29μm bearing egg cells. Since expression of PhII is induced by the sex-inducer protein (17, 35, 36), in stages I to IV with vegetative phenotype, it was added 24 h before microscopy to increase PhII expression; after such a short incubation with the sex inducer, the females show unchanged cleavage programs, unaltered growth dynamics and vegetative phenotypes (*SI Appendix*, Fig. S12). To obtain a changed cleavage program and a fully developed sexual phenotype (S), the females were sexually induced 72 h before microscopy.

### 1.3. Pherophorin II Is Localized in the Compartment Borders of Individual Cells.

**Fig. 4.**
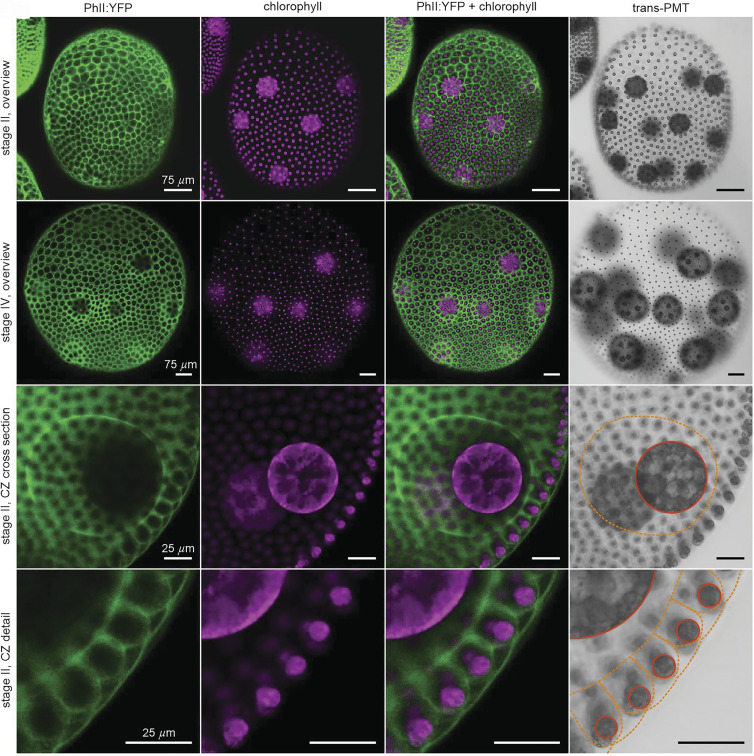
Localization of PhII:YFP in whole, middle-aged (early stage II) and old adults (stage IV) in top view and magnified cross section through CZ. Sexually induced transformants expressing the *phII*:*yfp* gene under the control of the endogenous *phII* promoter were analyzed in vivo for the localization of the PHII:YFP fusion protein. PhII:YFP is located in the CZ3 of both somatic cells and gonidia as well as in the BZ. Top views of whole organism: (*A*) stage II immediately before the first cleavage of the gonidia inside; and (*B*) stage IV before hatching of the fully developed juveniles. (*C*) Cross section through CZ in early stage II. (*D*) Magnified view of the outermost ECM region.(*A1*–*D1*): YFP fluorescence of the PhII:YFP protein (green). (*A2*–*D2*): Chlorophyll fluorescence (magenta). (*A3*–*D3*): Overlay of YFP and chlorophyll fluorescence. (*A4*–*D4*): Transmission-PMT (trans-PMT). PhII:YFP-stained ECM boundaries are highlighted in orange and cell boundaries in red.

As expected, PhII:YFP is only found in the extracellular space within the ECM. It is detectable at all developmental stages after embryonic inversion, which marks the beginning of ECM biosynthesis (34, 46), and in the ECM of organisms with the phenotypes of both vegetative and sexual development. At a first glance, in a top view, PhII:YFP appears to form a polygonal pattern at the surface of each postembryonic spheroid, with a single somatic cell near the center of each compartment (Fig. 4). PhII:YFP is also found in the ECM compartment boundaries (CZ3) of the gonidia, which are located below the somatic cell layer (Figs. 1*B* and 4). These observations hold at all stages after embryonic inversion, even as the shape of the compartment boundaries varies. Cross-sectional views of CZ3 compartments along the radial axis (Fig. 4*D* and *SI Appendix*, Fig. S13) prove that in all stages (I to IV) the CZ3 compartments are consistently arranged next to each other and in a single layer, with no deep overlaps or other 3D complications as seen in some epithelia (47). We discuss cross-sectional shapes of the somatic CZ3 compartments in stages I to IV and corresponding estimations of compartment volumetric growth in Section 2.7 and *SI Appendix*, Figs. S13 and S14 and Table S4).

**Fig. 5.**
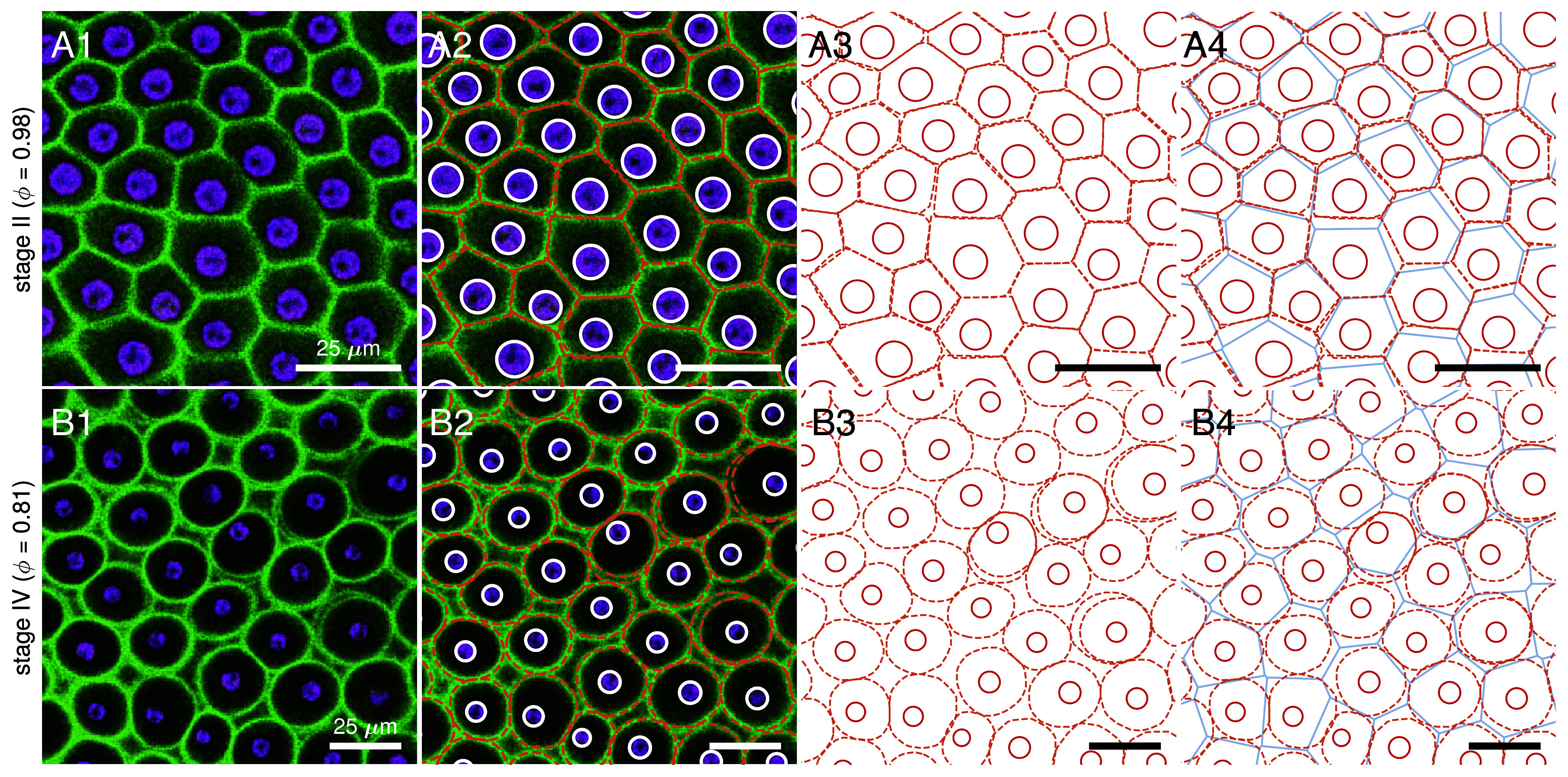
As in Fig. 4, but a close-up of PhII:YFP localization in top view. (*A*) Stage II. Magnified view of the somatic CZ3 compartments (1), identified by orange in column 2 along with cell boundaries (white), shown together in columns 3 and 4 with underlaid Voronoi tessellation (blue) and cell boundaries in red. (*B*) In stage IV the CZ3 compartments become more bubble shaped and individual CZ3 walls separate from the neighbors, leaving extracompartmental ECM space. Double-walls also appear, highlighted in *B3*. The surface covering fraction by compartments in these top view sections is denoted by ϕ, which decreases from approximately 100% in stage II (*A1*) to 80% in stage IV (*B1*).

On closer inspection in top view (Fig. 5), we see that: i) the somatic ECM compartments are a mix of hexagons, heptagons, pentagons, other polygons, circles and ovals, ii) the angularity of the compartment boundaries changes during expansion of the organism, i.e. the compartments become increasingly circular (less polygonal), and iii) each cell builds its own ECM compartment boundary. The observed localization of PhII is shown schematically in Fig. 12.

Especially in the early stages I and II, the fluorescent ECM compartment borders are predominantly pentagonal or hexagonal in top view (Figs. 4*A* and 5*A*) with a rough ratio of 1:2 (*SI Appendix*, Fig. S4). By stage IV they become more rounded and the interstices we term “extracompartmental ECM spaces” between the compartments increase in size and number (Figs. 4*B* and 5*B*). Since the compartment boundaries of adjacent cells are close to each other in early stages, the two compartments appear to be separated by a single wall. Later, when the boundaries are more circular, it becomes apparent that it is a double wall; each somatic cell produces its own boundary (Fig. 5 *A* and *B*). Fig. 5*A1*–*B1* show that the surface coverage of the CZ by compartments changes from ∼100% to ∼80% while the structure remains singly connected. The dynamics follow scenario Fig. 1*E* (ε): the CZ3 is geometrically coupled to expansion of the spheroid. This coupling forms the basis for interpreting aspects of development, such as the degree of AP differentiation (Fig. 4*A1*–*B1*), directly from CZ3 geometry, as we explore in Section 1.4.

As shown, PhII:YFP is a component of the CZ3 of both somatic cells and gonidia (Figs. 1*B*, 4, and 5). It seems to be a firmly anchored building block there, as the observed structure is highly fluorescent and yet sharply demarcated from other nonfluorescent adjacent ECM structures. If the PhII:YFP protein was prone to diffusion, one would expect a brightness gradient starting from the structures. There are no interruptions in the fluorescent labeling of boundaries associated with passages between neighboring compartments. As the boundaries are so close together in early stages, only the thickness of the double wall can be determined and then halved; we estimate stage II single wall thickness as 1.6±0.4μm and a similar value of 1.9±0.5μm in stage IV.

A comparison of the PhII:YFP-stained structures with descriptions of ECM structures from electron microscopy shows that the PhII:YFP location corresponds exactly to that of the ECM structure CZ3 (20). This applies to the entire period from the beginning of ECM biosynthesis after embryonic inversion to the maximally grown old adult.

**Fig. 6.**
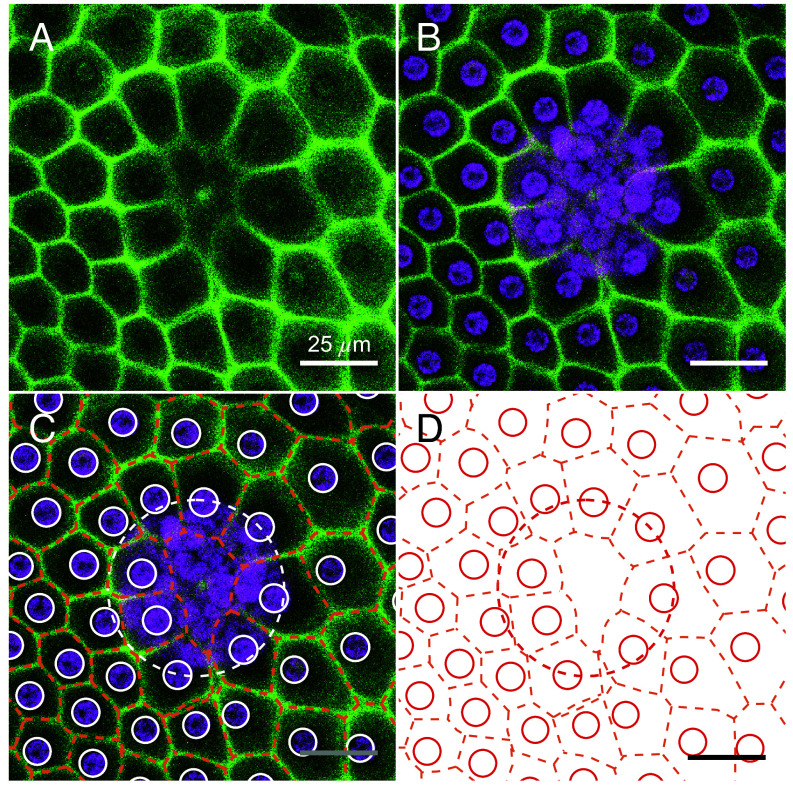
Magnified top view of CZ3 above a reproductive cell in early stage II. (*A*) PhII:YFP fluorescence (green). (*B*) overlay with chlorophyll fluorescence (magenta). (*C*) Overlay with highlighted CZ3 (orange) and cell boundaries (white). (*D*) as in (*C*) with only cell boundaries (red) and CZ3 (orange).

The relatively regular pattern of compartments (Fig. 5*A*) is disturbed by the gonidia (later embryos) which, being far larger than somatic cells, are pushed under the somatic cell layer. The CZ3 compartments of the deeper gonidia nevertheless extend to the surface (Fig. 6). The surrounding somatic CZ3 compartments are elongated in the direction of the gonidial CZ3 protrusions to the surface (Fig. 6). Since the PhII:YFP-stained CZ3 structure completely encloses each cell, these walls cannot be impermeable; ECM proteins must pass through them, as growth of the spheroid requires the cell-free areas outside the ECM compartments of individual cells to increase immensely in volume. This applies in particular to the deep zone, but also to the areas between the compartments. All ECM material required for this increase can only be produced and exported by the cells and must then pass through the ECM compartment borders of cells.

### 1.4. Quantification of Surface Somatic CZ3 Geometry.

**Fig. 7.**
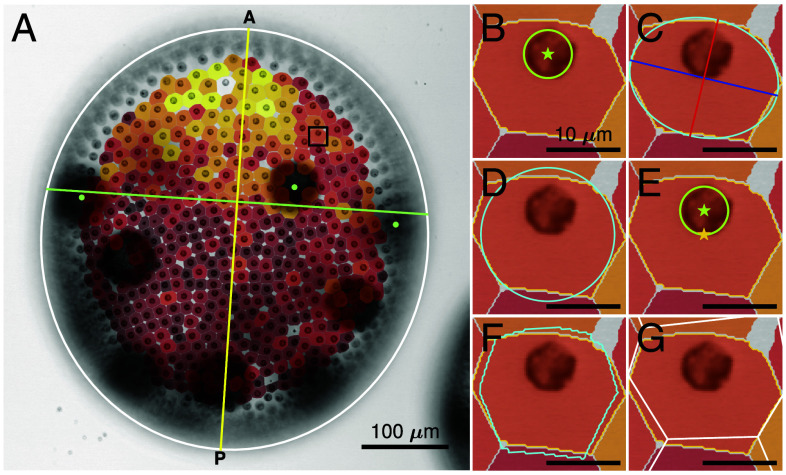
Geometric features of cell/compartments pairs. (*A*) Trans-PMT image of stage III spheroid in top view, with elliptical outline (white), estimated PA axis (yellow) that is orthogonal to line through gonidia (green dots). Overlaid are segmentations of CZ3 compartments, colored dark to light by size. (*B*–*G*) Schematics of geometric features computed from cell (green) and compartment (yellow) boundaries, as indicated: (*B*) $a_{\mathrm{cell}},a_{\mathrm{cz}3}$, (*C*) aspect ratio α and corresponding ellipse (cyan) with major and minor axes in blue and red, (*D*) deviation from a circle of the same area (cyan), (*E*) offset of cell area center of mass (green star) from compartment area center of mass (yellow star), (*F*) whitening transform of the compartment area (cyan), and (*G*) Voronoi tessellation (white) error $e_{V}$.

**Fig. 8.**
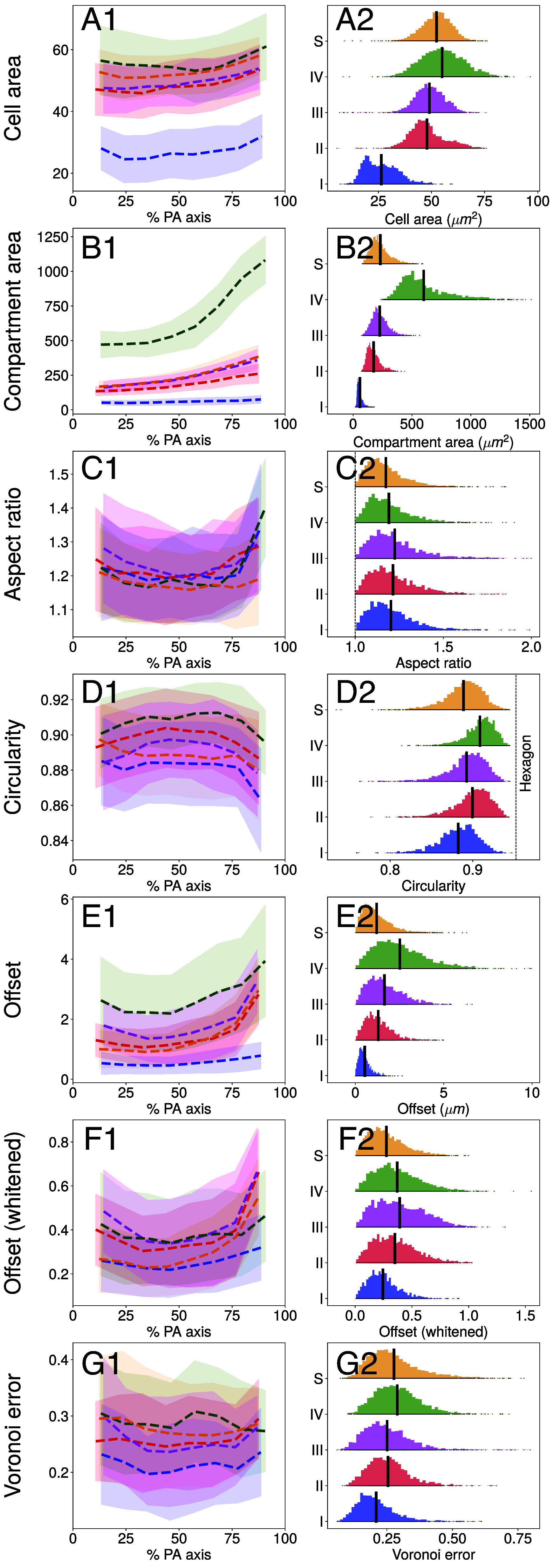
PA axis and life cycle variation in top view. (*A1*–*G1*): Computed metrics binned in 8 equally spaced segments along the PA axis. Means are shown as dashed lines with per-bin SD reported by shaded segments. Colors correspond to developmental stages defined in Fig. 3. (*A2*–*G2*): Histograms of metrics by stage in 100 equally spaced bins, by stage, with empirical means indicated by vertical black bars. Units are noted in parentheses, and otherwise, are dimensionless. See *SI Appendix*, Table S2 for numerical values of empirical means. *SI Appendix*, Fig. S15 and Table S5 show analogous data for distortion-corrected surface CZ3 geometry accounting for the mounting procedure (*Materials and Methods*). We find a maximum 5% increase in mean compartment area across stages I to IV, with minimal change in other CZ3 features.

**Fig. 9.**
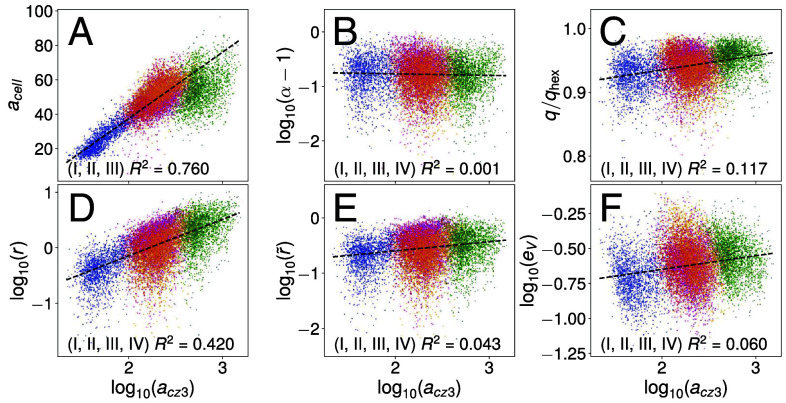
Pair correlations of compartment features in top view. At stages I to IV (blue, red, magenta, green) and S (orange), plots show correlations between (*A*) compartment area ($a_{\text{cz3}}$) and other metrics (*B*–*F*) defined in Table 1. Coordinate transforms are chosen in either linear- or log-scale, with natural offsets (e.g., $q_{\mathrm{hex}}$ in panel *C*, the circularity of a regular hexagon), to produce approximately equally sized contours across life cycle stages. $R^{2}$ is the linear regression correlation coefficient for stages listed. Similarly as in Fig. 8, geometric distortion-corrected versions of A, B, and C may be found in *SI Appendix*, Fig. S16. We find that the feature correlations are essentially unchanged, with a slight improvement in cell/compartment area correlation (*A*).

The localization of PhII at the CZ3 compartment boundaries allows us to carry out the first quantitative analyses of their surface geometry, both along the posterior–anterior (PA) axis and through the life cycle stages. A semiautomated image analysis (*SI Appendix*, section 2) reveals geometric features described in Table 1 and Fig. 7. A total of 29 spheroids across five developmental stages were analyzed: 7 in stage I, 5 each in stages II, III, and IV of the asexual life cycle, and 7 in the sexual life cycle (stage S). The mounting procedure (*Materials and Methods*) induces some elastic distortion in top view; we report here the original features, discussing distortion correction in the SI Appendix. The results below (Figs. 8 and 9) do not meaningfully differ with correction.

The PA axis of *V. carteri* spheroids is their swimming direction, and along this axis the distance between the somatic cells and the size of their eyespots decreases toward the posterior pole (Fig. 1*A*), as noted previously (6), p. 229]. Offspring (gonidia, embryos, and daughter spheroids) are mainly located in the posterior hemisphere. We approximate the PA axis by a line passing through the center of the spheroid and normal to the best-fit line passing through manually identified juveniles in the anterior (Fig. 7*A*). This estimated PA axis is typically well-approximated by the elliptical major axis of the spheroid (*SI Appendix*, Fig. S5).

The metrics shown in Fig. 7 (*SI Appendix*, section 2) are derived from the top-viewed compartment shape outline or from geometric moments of area. The matrix $M_{2}$ of 2nd moments

[1] $$\begin{array}{c} M_{2}=\int \int_{\mathrm{cz}3}\left(x-x_{\mathrm{cz}3}\right)⊗\left(x-x_{\mathrm{cz}3}\right)d^{2}x, \end{array}$$

and its normalization $\Sigma =M_{2}/a_{\mathrm{cz}3}$ are interpretable as elastic strain tensors with respect to unit-aspect-ratio shapes. The eigenvalues $\lambda_{\max},\lambda_{\min}$ (whose square roots define the major, minor axis lengths) of Σ are principal stretches of this deformation, yielding the aspect ratio and other quantities defined in Table 1. Overall, we measure changes in the moments of area (*SI Appendix*, section 2.B) to quantify ECM geometry during growth; the 0th gives the area increase, the 1st quantifies migration of compartment centroids with respect to cells, the 2nd gives the change in eccentricity (Table 1). The sum of second moments reveals changes in crystallinity of the entire CZ3 configuration, as described in Section 1.7.

**Table 2.** Summary of estimated volumetric growth by stage

Stage	Parent radius (μm)	Offspring radius (μm)	Somatic cell radius (μm)	Parental ECM volume change (est., mm^3^)	Parental ECM growth rate (est., mm^3^/h)
I	106±6	16±2	2.8±0.5	↓	↓
II	221±19	29±2	3.9±0.4	0.039	0.0026
III	244±13	30±4	4.0±0.3	0.015	0.0025
IV	422±6	79±6	4.2±0.4	0.229	0.0143
S	265±26	15±1	4.1±0.3	n/a	n/a

Values reported are mean ± SD. Estimated ECM volume is volume of spheroid minus that estimated of juveniles and somatic cells, as explained in *SI Appendix*, Table S1. Values in final two columns represent changes with respect to preceding stage.

### 1.5. Surface CZ3 Geometry Along PA Axis During the Life Cycle.

#### 1.5.1. Anterior CZ3 compartment areas expand toward end of life cycle.

Fig. 8*A1*–*A2* shows that somatic cell area increases modestly, by ∼10%, along the PA axis at all stages. In contrast, the CZ3 compartment area grows substantially along this axis, from ∼44% in stage I to ∼130% in stage IV (*SI Appendix*, Table S2). Moreover, the slope increases after the equatorial region in all stages, most prominently in stage IV (panel *B1*). Cell and compartment areas also increase by life cycle stage as shown in Fig. 8 *A* and *B*. Somatic cell areas double from I to II, growing merely ∼15% afterward, whereas compartment areas expand primarily after III, with a ∼160% increase occurring from III to IV (*SI Appendix*, Table S2).

Fig. 8*A2*–*G2* shows distributions of the metrics; apart from cell area, all exhibit positive skew and exponential tails which suggest good fits with gamma-type distributions (41),

[2] $$\begin{array}{cc} p_{\lambda ,k}\left(x\right) & =\lambda^{k}x^{k-1}e^{-\lambda x}/\Gamma \left(k\right), \end{array}$$

where x is suitably standardized. This skew should be considered when making mean-based comparisons across life cycle stages. The long left tails of cell area reflect the persistence of small somatic cells throughout the life cycle, confirmed by inspection in the chlorophyll signal. Last, the cell size distribution primarily translates rightward in time, while the compartment area size distribution simultaneously translates and stretches, indicating an increase in polydispersity.

#### 1.5.2. CZ3 compartment areas transition from tighter polygonal to looser elliptical packing.

Panels *D1*–*D2* in Fig. 8 show that while there is no apparent trend in the circularity of CZ3 compartment areas along the PA axis, the average circularity increases from stages I to IV. Since extracompartmental ECM space appears as compartment areas increase in circularity both effects correlate with enlargement of the spheroid. Fig. 8*D2* shows that circularity increases in mean while decreasing in variance, suggesting a relaxation process by which compartments of a particular aspect ratio but different polygonal initial configurations relax to a common elliptical shape with the same aspect ratio. This is also apparent by the ∼39% increase from stage I to IV in error with respect to the Voronoi tessellation (Fig. 8*G2* and *SI Appendix*, Table S2), whose partitions are always convex polygons.

#### 1.5.3. CZ3 compartment areas enlarge anisotropically.

While the compartment areas become more circular as they expand, the aspect ratio is independent of stage and thus of organism size. The apparent increase at extremes of the PA axis (U-shaped curves) is almost entirely accounted for by distortion correction (*SI Appendix*, Fig. S15 and Table S5), which still leaves the distributions (Fig. 8*C2*) unchanged. Fig. 8*C* shows that aspect ratio distributions are not only stable in mean, with less than 5% variation, but also in skewness and variance; they are gamma-distributed throughout growth with stable distribution parameters (*SI Appendix*, Fig. S9). Together, the stability of aspect ratio and increasing compartment area circularity during growth suggests a transition from tightly packed, polygonal compartment areas (where neighboring boundaries are closely aligned) to elliptical configurations in which neighboring boundaries are no longer in full contact. We term this process acircular relaxation.

To study how ECM is distributed around the somatic cells, we quantified the cellular offset during the life cycle. The absolute offset from the compartment area center of mass (Fig. 8*E1*–*E2*) increases from stages I to IV, and along the PA axis, indicating a strong correlation between larger compartment areas and cellular displacements (as quantified in Section 1.6). Perhaps counterintuitively, the cellular offset vector shows no correlation with the primary elongation axis of the compartment area; their relative angle is uniformly distributed in $\left[0,\frac{\pi }{2}\right]$ in I to IV and S (*SI Appendix*, Fig. S10). In contrast, the whitened offset (which accounts for compartment area and anisotropy) is nearly constant in mean after from II to IV (black vertical lines in Fig. 8*F*). The support of the distribution does increase, albeit at a smaller rate than that of the cellular offset. Throughout this analysis of variation along the PA axis (Fig. 8), similarly sized spheroids in the asexual and sexual life cycle stages, bearing embryos or egg cells respectively, resemble each other in ECM geometry.

### 1.6. CZ3 Geometry Shows Feature Correlations.

The analysis above indicates compelling correlations between geometric features of the CZ3 architecture during growth. Here, we analyze these in more detail with pooled data from all spheroids presented in Fig. 8. Fig. 9*A* shows an exponential increase in the compartment area $a_{\mathrm{cz}3}$ with cell area $a_{\mathrm{cell}}$ through stage III, saturating at stage IV. This quantifies the prior observation that somatic cells primarily grow before stage II, in contrast to compartment areas, which primarily grow after stage III. As expected from the PA analysis, the aspect ratio (B) is decoupled from compartment area size, while the circularity (C) increases. This reinforces the conclusion that as compartment areas expand they preserve their aspect ratio while decreasing in polygonality. The cellular offset (D) reveals a power-law relationship with compartment area, which, in conjunction with the weak coupling between whitened offset and compartment area size (E), further supports that conclusion in light of a scaling argument we explain in Section 2.5.

### 1.7. Tessellation Properties Change during the Life Cycle.

**Fig. 10.**
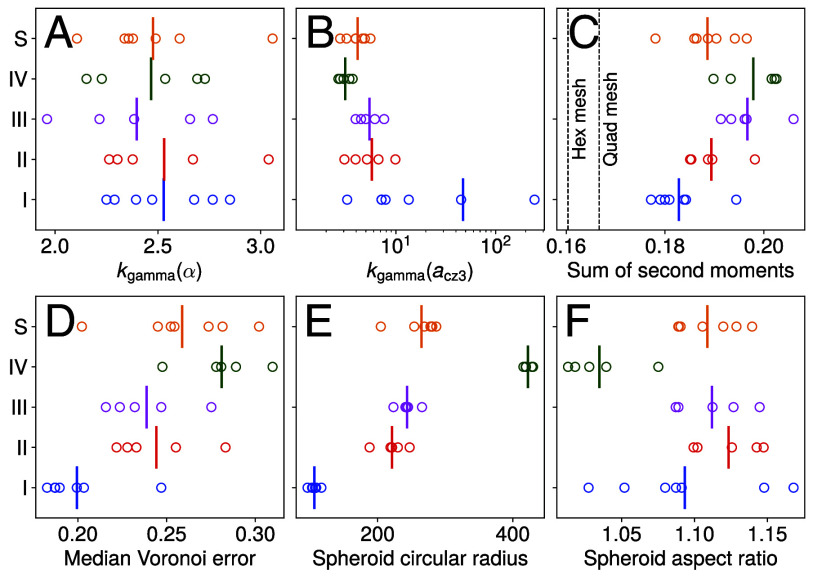
Global geometric properties of spheroids in stages I to IV, S in top view. (*A*) shape parameter k of gamma distribution fit to α, (*B*) the same for $a_{cz3}$, (*C*) sum of second moments Eq. **3**, (*D*) median $e_{V}$ over the whole spheroid, (*E*) the circular radius of each spheroid (geometric mean of major and minor axes), and (*F*) the aspect ratio of each spheroid (ratio of major and minor axes).

The metrics in Fig. 10 reveal clear trends by life cycle stage for the global geometry of each spheroid in top view. Panel *E* shows the increasing circular radius, with most of the increase occurring between stages I to II and III to IV. II to III is separated by fewer hours and occurs during the first dark phase. Stage S is sorted in size close to stage III, supporting its resemblance with stages II and III in the PA analysis.

At fixed mean, the shape parameter $k_{\mathrm{gamma}}$ of the gamma distribution, Eq. **2**, is a measure of the entropy of the configuration, with high k indicating an increasingly crystalline, Gaussian-distributed configuration by the central limit theorem (48). Fig. 10*A* confirms the stability of the aspect ratio distribution between stages, exhibiting values of $k_{\mathrm{gamma}}$ between ∼2 and 3, similar to ranges previously reported for confluent tissues (44). Simultaneously, panel *B* shows that $k_{\mathrm{gamma}}$ in the distribution of $a_{\mathrm{cz}3}$ is decreasing from stages I to IV, so the configuration (primarily the anterior hemisphere, *SI Appendix*, Fig. S11) becomes increasingly disordered. The initial high values of $k_{\mathrm{gamma}}$ are consistent with the earlier observation that CZ3 compartments begin in a tightly packed configuration, and as k quantifies regularity we infer that both tight packing and proximity to an equal-area lattice describes the initial configuration. The values of $k_{\mathrm{gamma}}$ between 2 and 3 in Stage IV are close to those for the Voronoi tessellations (41), which is remarkable given the decreasing polygonality of the CZ3 compartments observed in Section 1.5.2.

The standardized sum of second moments, defined as

[3] $$\begin{array}{c} n\sum_{i=1}^{n}\text{Tr}\left(M_{2}^{\left(i\right)}\right)/{\left(\sum_{i=1}^{n}a_{\mathrm{cz}3}^{\left(i\right)}\right)}^{2}, \end{array}$$

with i=1…n indexing CZ3 compartments per spheroid, is a space-partitioning cost that is minimized by equilateral hexagonal meshes (e.g., the surface of a honeycomb, see *SI Appendix*, section 2.C.2). Although CZ3 compartments do not tile space due to extracompartmental spaces, Eq. **3** can nevertheless be computed for the covered area. This energetic cost for each spheroid, representing deviations of the CZ3 architecture from optimal space-partitioning, is displayed in panel *C*. We find that the somatic CZ3 architecture becomes decreasingly optimal during expansion—an observation consistent with the underlying increasing trends in cell offset and area polydispersity as quantified in panel *B*, Sections 1.5.1 and 1.5.3. The metrics in *B* and *C* thus show the counterintuitive result that the CZ3 space partitioning is increasingly disordered as the global sphericity is maintained (and even improved, panel *F*) during the dramatic enlargement.

### 1.8. Pherophorin II Is also Localized in the Boundary Zone.

**Fig. 11.**
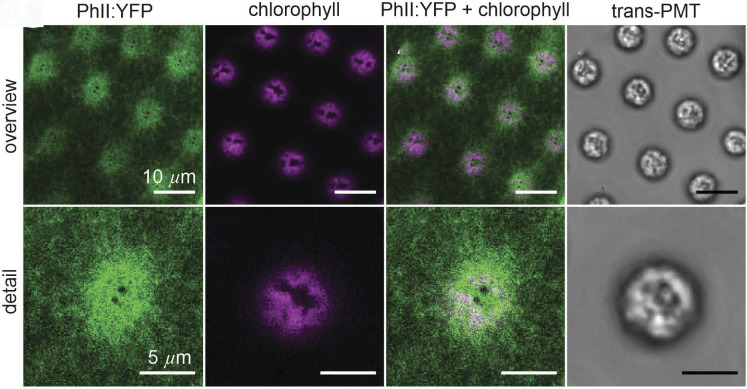
Magnified top view in regions where the BZ is connected to CZ3 in early stage II. Fiber-like structures radiate from these regions. Flagellar tunnels are seen in the centers of those areas as two dark dots. (*A*) shows an overview and (*B*) a detailed view. (*A1* and *B1*) YFP fluorescence of the PhII:YFP protein (green). (*A2* and *B2*) Chlorophyll fluorescence (magenta). (*A3* and *B3*) Overlay of YFP and chlorophyll fluorescence. (*A4* and *B4*) Transmission-PMT (trans-PMT).

Confocal cross sections reveal that PhII is also part of the boundary zone (BZ), the outermost ECM layer of the organism (Figs. 1*B* and 4 *C* and *D*). The PhII:YFP-stained BZ extends as a thin ∼1.1±0.4μm arcing layer from the flagella exit points of one cell to those of all neighboring cells. This shape indicates that the outer surface of the spheroid has small indentations at the locations of somatic cells, where flagella penetrate the ECM. At these points, the BZ is connected to the CZ3 of the somatic cells below. Because the BZ is thin and not flat, it is not visible in a top, cross-sectional view of a spheroid through the centers of the somatic ECM compartments (e.g., Fig. 5), and only partly visible when the focal plane cuts through the BZ. If the focal plane is placed on the deepest point of the indentations, only the areas at which the BZ is connected to CZ3 can be seen (Fig. 11). From the centers of these areas the two flagella emerge and the flagellar tunnels are seen as two black dots due to the lack of fluorescence there (Fig. 11*B*). A closer look at the fine structure at the BZ-CZ3 connection site shows that fiber-like structures radiate from there to the BZ-CZ3 connection sites of neighboring somatic cells.

## 2. Discussion and Conclusions

### 2.1. Holistic View of PhII Localization.

**Fig. 12.**
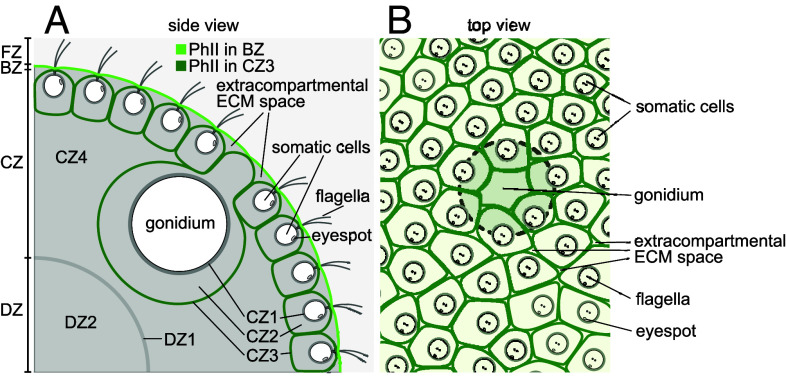
Overview of PhII:YFP localization in the ECM in early stage II. (A) Schematic cross section, showing localization in CZ3 of both somatic cells and gonidia (dark green) and BZ (light green). (B) Schematic top view, looking through the boundary zone, showing PhII:YFP in the CZ3 and the existence of extracompartmental ECM spaces. Position of a gonidium below the somatic cell layer is indicated (dashed); the CZ3 compartment of the deeper gonidium extends to the surface where it is surrounded by ECM compartments with somatic cells.

Synthesizing the results of preceding sections, we arrive at the summary shown in Fig. 12 of the identified locations of PhII. It forms compartment boundaries (CZ3) around each somatic cell and each gonidium, and is also found in the outer border (BZ) of the spheroid; CZ3 and BZ are connected where the flagella emerge. While each compartment boundary can be assigned to the cell it encloses, and is most likely synthesized solely by that cell, PhII in the BZ is evidently formed collectively by neighboring cells. Since the compartment boundaries of somatic cells are not completely adjacent to those of neighbors, and the BZ does not rest directly on the compartment boundaries, extracompartmental ECM space remains between the CZ3 enclosures as well as between them and the BZ. The extracompartmental ECM space thus appears to be a net-like coherent space connected to CZ4.

### 2.2. Relation to Earlier ECM Studies by Electron Microscopy.

In earlier transmission electron microscopy images showing heavy metal-stained sections of the ECM, both the CZ3 and the BZ can be recognized as relatively dark structures, whereas the CZ2, CZ4, and the deep zone are very bright (20). As the degree of darkness reflects the electron density and atomic mass variations in the sample, PhII evidently forms firmer wall-like structures in CZ3 and BZ, while CZ2, CZ4, and the deep zone have a very low density and are presumably of gel-like consistency. Using quick-freeze/deep-etch electron microscopy, it was shown that the fine structure of the ECM of volvocine algae such as *Chlamydomonas* and *Volvox* resembles a three-dimensional network (49, 50). While both the CZ3 and BZ are likely dense networks with a fine pore size to the mesh, they must nevertheless allow the passage of small molecules and noncrosslinked ECM building blocks exported by cells, as evidenced by the growth during development of these compartments, the extracompartmental space, and the deep zone. Cells must also be able to absorb nutrients from the outside, which must pass through both the BZ and CZ3. The BZ may be a denser network than the CZ3, in order to prevent ECM building blocks from escaping into the environment.

### 2.3. Mechanical Implications of Identified ECM Structures.

As revealed by the localization of PhII:YFP and prior electron microscope studies, the BZ appears to form a dense “skin” on the outer surface of the spheroid to which the CZ3 compartments are firmly attached at the flagella exit points. This point-like attachment allows the compartments to expand in all directions during growth. As the CZ3 compartments are densely packed and attached to the BZ, they would be expected to provide rigidity as a kind of “exoskeleton” of the alga. A simple experiment shows this feature: If many *Volvox* are pressed together inside the suspending liquid medium and then released, they elastically repel each other. And while it is clear that the constant expansion of the compartments by incorporating further ECM components allows the ECM to enlarge considerably, the precise mechanism for transporting the ECM components to their destinations remains an open question.

### 2.4. Evolution of the Volvocine ECM and Convergence of a Monolayer Epithelium-Like Architecture.

The ECM of *V. carteri* evolved from the (cellulose-free) cell wall of a *Chlamydomonas*-like, unicellular ancestor (7). The cell wall of extant *Chlamydomonas* species consists of an outer “tripartite” layer with a highly regular, quasi-crystalline structure and an inner more amorphous layer. In few-celled volvocine genera with a low degree of developmental complexity, such as *Pandorina*, the tripartite layer is partially split, so that its outer leaflet is continuous across the surface of the organism, while its inner leaflet still surrounds each individual cell body (51). In larger, more complex volvocine algae (*Eudorina* to *Volvox*), the entire tripartite layer is continuous over the surface of the organism. In the genus *Volvox*, the outer layer has developed even further and the tripartite layer has become part of the boundary layer, the BZ, while the inner layer has evolved species-specific (CZ3) compartments (20). The architecture of these CZ3 compartments shows certain parallels with the epidermis of most plant leaves (52) and even epithelia in animals (38), all of which possess an (initially) closely packed, polygonal architecture. Looking at the plasma membranes in animal epithelia, cellulose-based cell walls in epidermal cells of land plants and cellulose-free CZ3 structures of somatic *Volvox* cells as touching compartment boundaries, their packing geometry can be described using the same physical concepts (53). Interestingly, they also share the presence of an adjacent thin ECM layer, which represents a kind of boundary in all of them: the cuticle (secreted by plant epidermis cells), the basal lamina (secreted by animal epithelial cells), and the BZ (secreted by somatic *Volvox* cells). The shared geometrical solution likely represents an example of convergent evolution driven by the common pressure to evolve a monolayer epithelium-like architecture with protective and control functions.

### 2.5. Characteristics of the CZ3 Stochastic Geometry.

Examination of the stochastic geometry of the space partition formed by CZ3 walls, along with somatic cell positions, reveals four key findings. First, somatic cell growth occurs mainly between stages I-II, whereas CZ3 compartment areas in top view grow mostly during III-IV (Fig. 8). The surface areas of the compartments are well approximated by gamma distributions with anterior/posterior hemispheres exhibiting different values of k (*SI Appendix*, Fig. S8). Such area distributions arise in granular and cellular materials (42, 54); it is remarkable to observe them in intrinsic structures of an ECM, with nonstationarity in k revealing A/P differentiation.

Second, the aspect ratio distributions of compartments in top view are remarkably stable throughout the life cycle (Figs. 8 and 9) and are well approximated by gamma distributions with stable k (*SI Appendix*, Fig. S9). Maintaining a fixed aspect ratio α≈1.2 requires that each compartment area enlarges anisotropically, in strong contrast with the trend in aspect ratio of the overall spheroid, which is both lower and decreases from stages III to IV to less than 1.05 (Fig. 10*F*). This lack of local-global coupling suggests that compartment area anisotropy could be set by geometric constraints in the cellular configuration prior to stage I and may explain how the organism maintains (and increases) its sphericity despite the strong nonuniformity in size and shape of its compartments. Shape variability in the form of gamma-distributed aspect ratios arises in a large class of epithelial tissues and inert jammed matter (44) which exhibit deviations from optimal space-partitioning in the sense of Eq. **3**. The epithelium-like architecture formed by the CZ3 robustly falls into this class.

Third, the somatic cell offset from the compartment centroid increases steadily with compartment area (Fig. 8*D*) while the whitened offset remains relatively constant (Fig. 8*E*). Both observations suggest a growth-induced deformation in which space is locally dilated (in tangent planes containing compartment surfaces) as $R^{2}\mapsto \rho R^{2},\rho \geq 1$, which indeed increases offsets while preserving whitened offsets (*SI Appendix*, section B.1). Such transformations also preserve aspect ratios, consistent with earlier observations. The cellular offset angle with respect to the principal stretch axis, on the other hand, is a priori unconstrained by these observations, and we find it is uniformly distributed in [0,π/2] (*SI Appendix*, Fig. S10). This highlights a stochastic decoupling between cell positioning and compartment shape, much like that previously observed between compartment and spheroid principal stretches. Likewise, this may be established before stage I and later scaled by compartment growth—analogous to two points diverging on the surface of an expanding bubble. Importantly, global dilations of space, likening *Volvox* itself to a bubble, cannot produce the increasing compartment area polydispersity observed during the life cycle, while local dilations as above, likening the CZ3 “epithelium” to a spherical raft of heterogeneously inflating bubbles, can. In a continuum limit, such growth-induced local dilations may be represented by conformal maps (55).

### 2.6. The CZ3 Structural Transition.

As best seen in Fig. 5, the CZ3 compartment mesh undergoes a structural transition (in top view) from a space partition to a packing whose surface covering fraction decreases significantly while remaining a connected structure. ECM-filled extracompartmental spaces expand during this transition, accompanying an apparent relaxation of the compartments from polygonal to elliptical shapes. This is reminiscent of the wet-dry transition in two-dimensional foams, wherein initially polygonal bubbles meeting at Plateau borders relax under surface tension to circular shapes as the liquid fraction (here, extracompartmental space) increases. Convergence to spherical bubbles and loss of contacts occurs at the jamming transition, which for 2D foams occurs at gas fraction $\phi_{c}\approx 0.84$ (56). While a systematic investigation of the average contact count and covering fraction ϕ of compartments requires comprehensive extraction of features from full 3D data (accounting for defects induced by offspring, Fig. 4*B1*), our preliminary results from top view (Fig. 5) indicate that the covering fraction can decrease to as little as ϕ≈0.81 in stage IV. Viewing the surface CZ3 geometry as a 2D foam, then, it is perhaps surprising that the aspect ratio distribution remains so stable (Fig. 8*C*) with mean α≈1.2 given that ϕ is near the jamming transition. On the other hand, a foam model for the compartment geometry may appear reasonable given the dramatic shape changes during development, but foams are vanishing-adhesion limits of more general plausible models (57) used previously for cell monolayers. There, cell–cell adhesion produces aspherical equilibria even at low ϕ; indeed, here, intercompartmental adhesion is likely stronger than that of foam bubbles due to crosslinking between the walls. Cross-sectional views (*SI Appendix*, Fig. S13) confirm that walls maintain strong radial contact throughout expansion.

More broadly, while structural transitions during development [including not only jamming (44, 57, 58) but also density-[ϕ-] independent rigidity transitions (59)] have been widely studied in epithelia, we now observe such a transition in the ECM of *Volvox*. This perhaps paves way for yet another usage of *Volvox* as a model multicellular organism: for the development of epithelium-like structures and transitions in ECMs and bioinspired materials. These are necessarily self-assembled structures, dependent on material properties like tension, adhesion, and permeability that are not under direct control by cells. This highlights more broadly the need to probe the rheology of the ECM—perhaps using microrheological techniques like those applied in cytoskeletal studies (60)—to understand how stochastic local interactions give way to robust global structures. A better understanding of the self-organization of crosslinking ECM components will shed light on the principles underlying the intricate geometry and stochasticity observed in multicellular ECMs.

### 2.7. The Shape of ECM Compartment Boundaries Along the Radial Axis: An Outlook.

In this study, we analyzed the surface features of the CZ3 in top view, which provides insight into the ECM packing geometry at the spheroid’s expanding surface. We also provide preliminary data on cross-sectional shapes (*SI Appendix*, Fig. S13), with the caveat that “floors” exhibit diffuse or poor YFP signal. From this, we estimate compartments’ volumetric growth and find that they indeed increase from stages I to IV, though nonlinearly, showing a sigmoidal growth (*SI Appendix*, Table S4 and Fig. S14). Moreover, the shape itself shows notable dynamics: while appearing roughly self-similar from stages I to III, it abruptly reverses its cross-sectional aspect ratio from III-IV from being slightly radially elongated to significantly tangentially elongated (*SI Appendix*, Fig. S13). This could indicate a transition in the growth dynamics at III to IV in which the boundary and deep zones are expanding faster than the cellular zone, with the latter building residual stress as a result. This picture of increasingly pressure-driven growth of the CZ (in which it is pulled apart laterally like the elastic surface of a balloon) is also weakly supported by observations of the spheroid’s eccentricity (Fig. 10*F*) sharply decreasing toward sphericity from III to IV. The full 3D dynamic geometry of the ECM is the subject of future study.

## 3. Materials and Methods

### 3.1. Strains and Culture Conditions.

Female wild-type strains of *V. carteri* f. *nagariensis* were Eve10 and HK10. Eve10 is a descendant of HK10 and the male 69-1b, which originate from Japan. The strains have been described previously (61–64). Strain HK10 has been used as a donor for the genomic library. As a recipient strain for transformation experiments, a nonrevertible nitrate reductase-deficient (*nitA*^−^) descendant of Eve10, strain TNit-1013 (65), was used. As the recipient strain is unable to use nitrate as a nitrogen source, it was grown in standard *Volvox* medium (66) supplemented with 1 mM ammonium chloride (NH_4_Cl). Transformants with a complemented nitrate reductase gene were grown in standard *Volvox* medium without ammonium chloride. Cultures were grown at 28^°^C in a cycle of 8 h dark/16 h cool fluorescent white light (67) at an average of ∼100 μmol photons m^−2^ s^−1^ photosynthetically active radiation in glass tubes with caps that allow for gas exchange or Fernbach flasks aerated with ∼50 cm^3^/min of sterile air.

### 3.2. Vector Construction.

The genomic library of *V. carteri* strain HK10 in the replacement lambda phage vector λEMBL3 (68) described by Ertl et al. (27) has been used before to obtain a lambda phage, λ16/1, with a 22 kb genomic fragment containing three copies of the *phII* gene (28). A subcloned 8.3 kb *Bam*HI-*Eco*RI fragment of this lambda phage contains the middle copy, the *phII* gene B, used here. The 8.3 kb fragment also includes the *phII* promoter region, 5’UTR, and 3’UTR and is in the pUC18 vector. An artificial *Kpn*I site should be inserted directly upstream of the stop codon so that the cDNA of the *yfp* can be inserted there. This was done by cutting out a 0.5 kb subfragment from a unique *Mlu*I located 0.2 kb upstream of the stop codon to a unique *Cla*I located 0.3 kb downstream of the stop codon from the 8.3 kb fragment, inserting the artificial *Kpn*I with PCRs, and putting the *Mlu*I-*Cla*I subfragment back to the corresponding position. The primers 5^′^GTAACTAACGAATGTACGGC (upstream of *Mlu*I) and 5^′^*ATCGAT*TCAGGTACCTGGCCCCGTGCGGTAGATG were used for the first PCR and the primers 5^′^GGTACC**TGA**TTGCCGTAAGAGCAGTCATG and 5^′^TCTAGCCTCGTAACTGTTCG (downstream of *Cla*I) for the second PCR (The *Kpn*I site is underlined, the stop codon is shown in bold). One primer contains a *Cla*I (italics) at its 5^′^ end to facilitate cloning. PCR was also utilized to add *Kpn*I sites to both ends of the *yfp* cDNA. In addition, a 15 bp linker sequence, which codes for a flexible pentaglycine interpeptide bridge, should be inserted before the *yfp* cDNA. The *yfp* sequence was previously codon-adapted to *C. reinhardtii* (69) but also works well in *V. carteri* (11). Since this *yfp* sequence was already provided with the linker sequence earlier (70), the primers 5^′^GGTACC*GGCGGAGGCGGTGGC*ATGAGC and 5^′^GGTACCCTTGTACAGCTCGTC and a corresponding template could be used (the *Kpn*I site is underlined, the 15 bp linker is shown in italics). The resulting 0.7 kb PCR fragment was digested with *Kpn*I and inserted into the artificially introduced *Kpn*I site of the above pUC18 vector with the 8.3 kb fragment. All PCRs were carried out as previously described (71–73) using a gradient PCR thermal cycler (Mastercycler Gradient; Eppendorf). The final vector pPhII-YFP (Fig. 2) was checked by sequencing.

### 3.3. Nuclear Transformation of V. carteri by Particle Bombardment.

Stable nuclear transformation of *V. carteri* strain TNit-1013 was performed as described earlier (74) using a Biolistic PDS-1000/He (Bio-Rad) particle gun (75). Gold microprojectiles (1.0 μm dia., Bio-Rad, Hercules, CA) were coated according to earlier protocols (71, 72). Algae of the recipient strain were cobombarded with the selection plasmid pVcNR15 (76), carrying the *V. carteri* nitrate reductase gene, and the nonselectable plasmid pPhII-YFP. Plasmid pVcNR15 is able to complement the nitrate reductase deficiency of the recipient strain and therefore allows for selection of transformants. For selection, the nitrogen source of the *Volvox* medium was switched from ammonium to nitrate and algae were then incubated under standard conditions in 9 cm diameter petri dishes filled with ∼35 mL liquid medium. Untransformed algae of the recipient strain die under these conditions due to nitrogen starvation. After incubation for at least six days, the petri dishes were inspected for green and living transformants.

### 3.4. Confocal Laser Scanning Microscopy.

For live cell imaging of transformed algae, cultures were grown under standard conditions and induced with 10 μL medium of sexually induced algae in a 10 mL glass tube. Approximately 10 spheroids in culture medium were placed on a glass slide and covered with a glass coverslip. The coverslip was mounted on grease on all four edges in order to apply exactly the right force to hold the spheroids in place but minimize deformation. Intact spheroids with an average number of gonidia, embryos, or daughter spheroids were selected for imaging. From all acquired images with stable spheroid positions, optimal focal planes were selected for segmentation and downstream analyses. An LSM780 confocal laser scanning microscope was used with a 63× LCI Plan-Neofluar objective and a 10× Plan-Apochromat (Carl Zeiss GmbH, Oberkochen, Germany). The pinhole diameter of the confocal was set to 1 Airy unit. Fluorescence of the PhII:YFP fusion protein was excited by an Ar^+^ laser at 514 nm and detected at 520 to 550 nm. The fluorescence of chlorophyll was detected at 650 to 700 nm. Fluorescence intensity was recorded in bidirectional scan mode for YFP and chlorophyll in two channels simultaneously. Transmission images were obtained in a third channel by using a transmission-photomultiplier tube detector (trans-PMT). Images were captured at 12 bits per pixel and analyzed using ZEN black 2.1 digital imaging software (ZEN 2011, Carl Zeiss GmbH). Image processing and analysis used Fiji (ImageJ 1.51w) (77). To verify the signal as YFP fluorescence, spectral analysis was performed. The lambda scan function of ZEN was used in which a spectrum of the emitted light is generated by a gallium arsenide phosphide QUASAR photomultiplier detector that produces simultaneous 18-channel readouts. Emission spectra between 486 and 637 nm were recorded for each pixel with a spectral resolution of 9nm using a 458/514 beam splitter and 514-nm laser light for excitation.

## Supplementary Material

Appendix 01 (PDF)

## Data Availability

Genetic sequences, images, and software data have been deposited in Zenodo (78).
