# Supplementary material for: Spatiotemporal distribution of the glycoprotein pherophorin II reveals stochastic geometry of the growing ECM of Volvox carteri
Source: Proc Natl Acad Sci U S A. 2025 Aug 12;122(33):e2425759122. doi: 10.1073/pnas.2425759122 (PMC12377771; doi:10.1073/pnas.2425759122)
Supplement: Supplementary file 1 — Appendix 01 (PDF) [file pnas.2425759122.sapp.pdf]

PNAS

Supporting Information for

Spatiotemporal distribution of the glycoprotein pherophorin II reveals stochastic geometry of the growing ECM of *Volvox carteri*

B. von der Heyde, A. Srinivasan, S.K. Birwa, E.L. von der Heyde, S.S.M.H. Höhn, R.E. Goldstein, and A. Hallmann

Corresponding Authors: Raymond E. Goldstein and Armin Hallmann
E-mails: R.E.Goldstein@damtp.cam.ac.uk and armin.hallmann@uni-bielefeld.de

This PDF file includes:

Supporting text
Figs. S1 to S17
Tables S1 to S5
SI References

Supporting Information Text

1. Supplementary data: Pherophorin II overview and DNA sequences

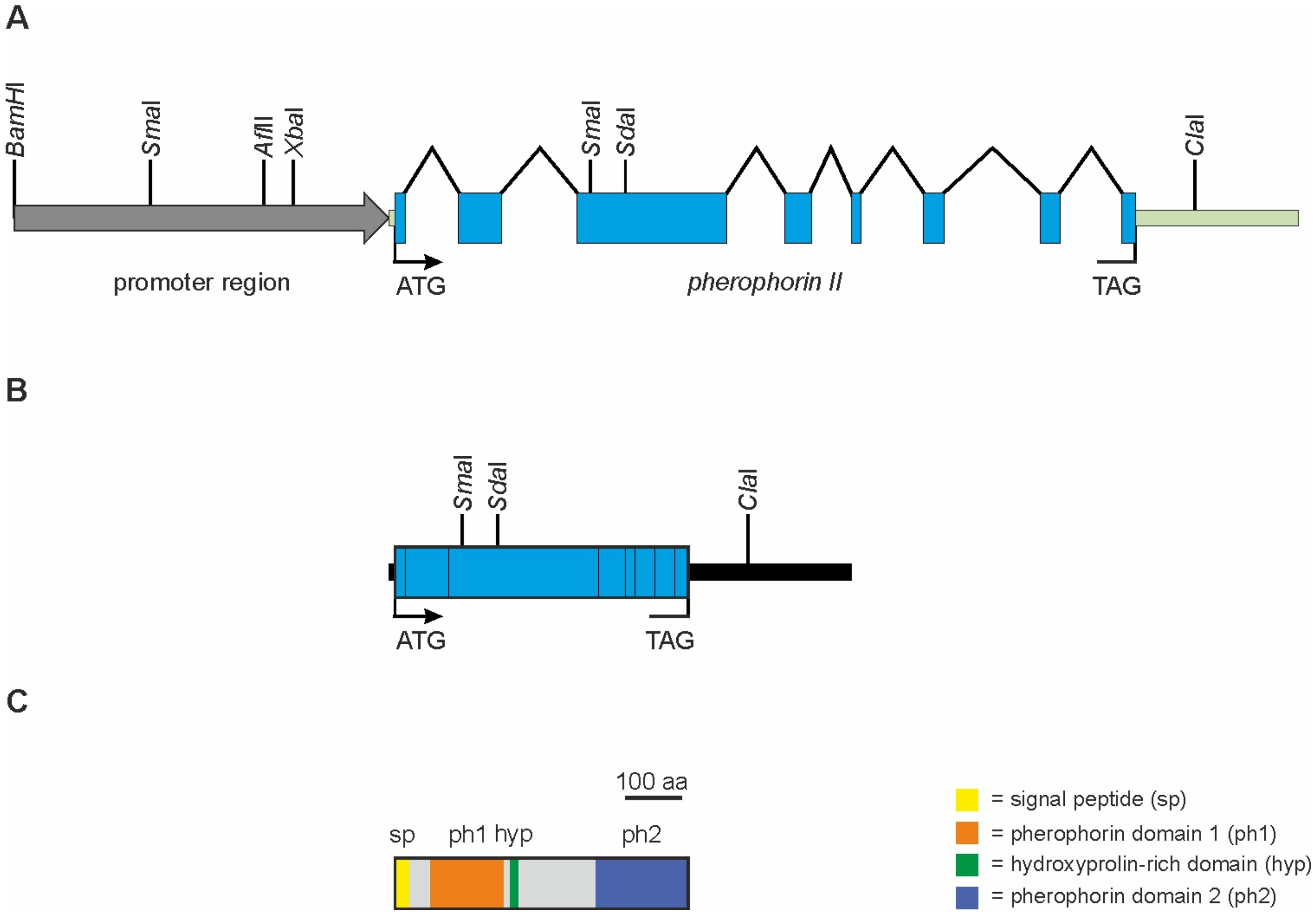

Fig. S1. Schematic structure of the *phII* gene, *phII* mRNA and pherophorin-II protein. (A) The genomic region schematized here corresponds to the 8329-bp genomic fragment utilized in plasmid pPhII-YFP. The *phII* gene (1) is located on scaffold 34 (nucleotides 980223 to 985045) of the *V. carteri* genome version 2.1 (2) in Phytozome v13 (3) on the reverse strand. The start codon is at nucleotide position 985025-985027 on the reverse strand. In the current *Volvox carteri* genome annotation available at Phytozome v13 (Volvox v2.1) pherophorin II is not annotated. Therefore the gene structure was established based on older annotations and confirmed with RNA-Sequencing data (4). The gene structure is indicated as follows: Coding sequences are represented by blue squares, intron sequences by carats, UTRs by green bars and the promoter region by a grey arrow. Start (ATG) and stop (TAG) codons are highlighted. The given restriction sites are also marked in SI Appendix, Fig. S2, which represents the genomic sequence of the *phII* gene. (B) Structure of the *V. carteri phII* mRNA. Sequence features are as indicated in A. The coding sequence (blue squares) totals 1557 nucleotides. The 5' UTR is 18 bp in length, while there is a quite long 3' UTR of 869 bp. The complete mRNA is 2,444 nucleotides in length. (C) Structure of the *V. carteri* pherophorin-II protein. The polypeptide comprises 518 amino acids and the calculated molecular weight amounts to 54.5 kDa. As pherophorin-II is an extracellular protein, it possesses a cleaved N-terminal signal peptide (sp) of 24 amino acids. In the mature protein three domains can be identified: an N-terminal pherophorin domain (ph1) with an E-value of 4.5e-27, a hydroxyproline-rich domain (hyp) in the middle (5) and a C-terminal pherophorin domain (ph2) with an E-value of 2.1e-32.The pherophorin domains where identified by blasting the Pfam database (6) using the hmmscan function (7). The short Hydroxyproline-rich domain consists of 78% (hydroxy) prolines (seven of nine amino acids).

GGATCCATGACTGGAAAACCCATCCATGACCAAGTCGTTCCCCGAGAGATAGCAGCTTTGGATCGACT *BamH*I
CGTCGCTTGTGAAAGCCTACCTGGTTACTTAGACGGATTTAGCGACTAGACCACTTATGAAGGTGTTG
ATAAACACCGGGGGTTTCCCTTGGGGTTTTATAGTCGCTTGCAGTCCGTCTGAGGCGCTCGGGATAAT
ATGCCGTACAGTAGTAGATATTCCTGCAGAAGAGAGTCGCACCTCCAGGGAAGAGTTACAATACAGGT
CTGGCCGTGAACGGGCAGACGTGTACAAGATGGTGTACGGAATCGAGTATGTGCACGGATCGTTACAG
CCATGGCCGTAGCCATGTACTGCTGAGTACGGCTGTAGTCGAAAGATGACGAAGATAAGATAGTTTTA
TGTACTGTGTGTACGATATTCGCCTCGACGATGGAATGATAAGAAGGAATGGAGTCCCTACTCGGAAG
TCCTGAGCCAGGCTCTTACACTGGGCACACATTGCCATCCAATGGATGGCCATGTGAATCACAGTGGT
GCCCGGCCTAAGGTAGCTAAGGACTTGGCCGTTATCATCGGTAGATTGCTCACCCACCTTCTCCGACC
AGGAAAACATCCTCGTCACTGGTGCACAGCTCGTCATAGCGATGAATGTTGCAGGGGTGCAAAGGCAA
GTTCCACCACACCATTATGAGCAACCTTAGGACCGCGTTCC**CCCGGG**TAGACACAGGAGGTTTCAGGG *Sma*I
GCTAGATATACTGGTCCAATAAGCAGGGTGCTATTGATGTAGGCAGTAATGTAGCACCCTCCCTCATC
CGACGTACCTCACTCCCTGCACATGTAGAGCCCCTGCGTAGAACGGCTATCTGTGATGAGGGGGAGTC
CCTGGTATAGAGGCCAATGGGAGCATGTGCCGTTGTTGGGCATTCGAGGGGTGCGTGACAGCGTTGTC
ATCGGTAGGGTCGTGCCGAGTTGTGAAGACGGGCTCCTGTAATGGGCCTTATCATGCAGTAGGTTTCT
TGTGGTTTCCGAGGCCGAGGTCATTTTGCCGGGGTGTACGGCATTGTTGCGGGTTGCGGGAAGTTGTA
CAGGTAAATGCATCGGCCATTTTTATCAGCCTTGTTTAAATATAAGTAGACAACCGAGCAAGAACCAA
AGTTTGTCGGAAAGAATTTGGGCAGAGGCCGAAGCTCGCCTGGCAAGCACTGCGCTGAAGGGATAGAG
AGAGACACGAATAAGAAGTGTACAACGGGGCCTCAATAGGCTTTGGGGTCCAGATCGCTGACGCCCTC
GCTCTCAGGCATCAAGTCCCGGACATGGGC**CTTAAG**ACCTGGAGTTGCTGGGCTATTCCCTCTCGCCC *Afl*II
TCCCCGCTCTGACCACTATCCCTCTCTCCAGTTCCCTTTCAGTTCGGGTGGTGCAACGCCAGGCCCCT
CGCCACACACACCGCACCGCACCCACGTTTGTTAAGCGCTAGCAGGCTGTTG**TCTAGA**GCTGTAGAAG *Xba*I
TTCCTAGGTCTATGTTGATGTAGTTTGGTCCCGTTGCTGATGCAACTGGCTTTCGCATCATTAACCGC
CGAGTAGCAGCCGTAGCTACCGGTGCGGAAAGGATGCATGCTCACCAACCGAAATGATGAAGTGCGTG
TGGCGAAGTTATATTCATTATGCCAGGATCAGAGATGTTGGTCAGTAAATAGCAAGTTATATAAAAGG
AATGGTGCTACAGATTACAACGACGGGTCGGATTCCGAAACGTACAACACGAGCAACATCCCAACGGA
TGAAATGCACATTTATATGTGTCCGCGACTAAC**GAATTC**TGCGCTGTGTTTCTAAGATTTATATTCTG *Eco*RI
CATAAAGATATTGATTCCAAAGGACGAAGCGCCTCTGTTGCGGTGGCCAGTCTCGGAAGCGGCTACCT
GCGCCCGCCGTTTACGGTAGTCGTACGGCCCCGCAGCAGCACAGCTGCCGCGGTCGGTCGGGTAGATA
AATAGCGCCACTATGATACAGAACATCATCAAAGTGACATAGCTCTGACAAT**ATG**GCATACTTTGCAA start
AGGTAGCTTTCATTTTGGCGACAGCGCTGGCGGTTGGCTGGTGCGTTTTTAGCATGGTGCATTCCCAT
ATGCCTGGGCTTACAATCTGAGGTTGGGTCTATATATTTAAGTATATTTAAGAGCCATATACTTGAAG
GCTTTCAGTCTTTGCCTTTGCGTCGCAAAAGACGTACATGTCCTGTCGCATGCCATAGTGATGAGCCT
TTTCTATAAAGCAATACATAAAAGATAAGACGTCTACAATCATAACTCGATCATGAAACAGCAAGCGT
CAACCATGAGCACTGACCTTGCTTGCTGCTTGTCGCCGCTGGTTCCAGGGCGCTCAGCGCTCATGCGC
AGGAATACAACGAGTACGATCCCTCAGCTGTCACGCCTGGCAGCATCCCCAACTTCCCATTCCGCGAC
TGCAACACCACAAACGGCGCGTACCGGCTGGCACCGGTGTGGCGGCCTTCGGGCAGCAACACGTACTG
CTTCAAGATCCAGGTCAGCCAGGATGCCCTGTCCTGCACTGGCGCCTGCTGCAGCGCCGACTTGCACA
AAATTGAGGTGAGCCCACGGCCGGCTGATGGTGTGGGGGATGCGACATGCGGACACAGTGCTACAGGT
CCTAGGGCCTCGGAAAGCACGGGAAGAGGCTCATTGGGCCCGCAACCAAATGTCAACGATTATTTTCC
AGTAGTTTCTCATGTGTGTGTGTGTGTGCATGTGCGCATGATGTCGGTGTTGAGGGCTCCGTTACCAT
CCTAGCTGCTCCTCCGGGGAAGTGGATTTCTAATCAGGGTTCGGAACGCGCCTCACAATATTTACATA
GCTCATTTACATGTGCTGGAGAGAATGAGAAGTTCGCACCCTCAAAAGTAGAGGGAACATCAAATTGT
GATGTTCCCTCCTGCCTCACCTTGCCCTTACTCCATCCTCCCACCACATGCCACCCCGCCATGTCCGC
AGTTCAACGTGTCCAGCAGCTGCCTGGTGGCCGGGGCCTCAGTTGTCGCCACCGTGAATGGAGTGCGG
A**CCCGGG**TTGGTGCGACCCTGGACAAGGCCCCGGCTGGGCCTGTTGGCTCCGCCGTTCTGCGGCTCAC *Sma*I
CCAGCTGGGGCTGGACACCACGACCGCCCAGGATGCGGAGGTGTGCCTCACCCTCAAGACCAACCGCG
GAGGCCAGGGCTGCACCACCCTGGAGCAACTGTGCTCGTCGCCAGGCTTCCCTGCAGGGACATGCACA
GCAGCTATGTTCGATGTCGCGTGTGATTGCTGCCCGGTTTCTCAAGCCGGTCAGGCCCTCCCGCCGCC
GCCACCAGTTATTCCGCCGGTCTTCCGGCCTTGTGACGTCTGCATCGCCGCGACCATTGTGCCCCCGG
CCAATGATGTGCGACCATACCGCTACGACAGCGCCACCTGCGCCGCCATTCAGCAGAACATCGCCGAT

GCCATGAACTCTCTCCTGGGAGGCGCCAACATCGGCGCCTTCTCACCTTTCGCGCCAAATGCAAGCCA
GTGCTTTGACACACAAATCATTACATGTGGCAGGTTCAACGGTTCGGACAGTGATGCGTTGGCCAATC
TGACGGAAGCAGTGCAGCAGCAGCTTTCCGCCTTCATTGGAGTCGCGTCTGGCGGCAACGTCTGCAAT
CCAAAGTTGGAGAAATACACGGTGACGGTCTCCACCATCAACAATGTAGCAGATCAGTGCCTGGACCT
CTCGCAGTCGGCTAGTTGCTTCCTGCCTGGCGTTCCATTCCCCAACTGCACGTGAGTCACCCTACTTC
ATGGCGTCGTCTCTTTTGCGGCGCGGCCGCCCTCCGTAAAGATTTGGGGGCGAGGATGTAGGGCCTAA
TAACCAAAGCTTCCCCCCGCAATGGATGGACCCATGTTTGGGATCTGGCTAGCGTCTTTGTCGTGTAT
GCAGAGCTTTGGATGAATGGCTGAAGGCAAAGACACCGCGCTTGCATGGATACCTTGCTCACATGGCT
ATTTGAAAAAATCAGGAACGTTGGGTGAATATGTGATAGTACTAGTTACCGACCCTGCTGCCGCCTCC
GCTCGCCCTCTGACGCGCAGGTGCAACACAACCCAGGGAGTCATGCCGTTCACAGTCTCCCCCACCTG
GTATGCACAGCCGGCCAATGTGCGCTGGGGCCGCAACGTCACTGAGTACTGCTTCACCGTCAACACGC
TACAGCCAAGTCAGGTCGTGCCGGTGAGGCCAGATAGGTGCATGGAGGACCCGTGGGGTGGCGCTTTG
TTTTCTTCCACGCACCCATGCATGCTTGGCTGCATGCCTGATGAGCTCGGTTAGTTGTATCGGGTCTG
GACAAGGCACACGGGCCTGTCTGCTGCGCGTAGCTCACCCAGACAAAGTTAAGAAATTACATGACTGA
GGATGCTGCCTCGTTCGCTGTCCCCCTGCAGAGCACCTGCTACAACGCAAACGACGCGCTCGCCAAGA
TCGAGTGGTATGCAAGTAAGTTATCATTGCTGTTGCTTGCTTGCGTAGTCTTCTATCGTTTCAACGTT
TCTTAGATAAGACCCGTCCTTTCAACCTGGGCAGACATGCATGGATATGTGGGAGTACGGCATGCGAA
TGGATATGGATATGTGCAAACTTCTACTGTCGCAGCATGATGTGAACTCTGGCCCTGCCGGCTGCACA
CATACGCATACATGACTTCCGTAATGCGAGCAGACTGCAGCATAGCACCCAGTACGGCAGCATCAGTG
GTTAACCTTTTTTGGTCCTCGCTACAGCCTAGGGTTTCACCTGCCGTGCATTTTGGCTTGTGTTTCCA
TGACTGCAGGTGATGCGTTCAGGTCGGCGGTCAAGGGTTTCACGGTGTACCCCGCCGGGGGATCAAAC
AAGACCATTGCCGACAGCTGGGGCGCCACTGGAACCGATACCCTGAAAGGTGAGGCCGGCAAGCTAGA
TAGCAGCTCAGGTAGATGCACCTCGTGCGGGGCTCATTCGCTGCTTCGATTGTGGATTCTGTGCGGCA
CTTACCTGCCGTGTGTCACAAGGGTAACTGTCGTGCGAGACCTTCACTTACAATACACTCGAGGAAAT
CTCGACGGACCTGCATCTCGGAAGGCCTCTTCGATGACCCTATCAAAAGATCAGCAATAAGCTCCAAC
AGTGTATGAAGATTGACGGAGCTGTCAAGCTCAATAACATAATCCCGAAAATGGCAAGCACTACGGCT
ACTGTATAAAACCCAGCGCAGAGGCTACACTCCCTTCGCTCCTGGCGCATCTAAGCTGCAATTACAAT
TACAGTAACTCTGTTCAGTAGCCAGCCATGCGCCGGTTTGTTTGTACCGTACCTGTCCAATTGCCATG
TGCTGCACCATCACTGCTAATGCCTGCATGTCAAATAAGATCACATCGTTCCCTGCCTGCGCATCCTT
TTGTTCGTGTGTGCAGTCAATCTCAACTGGAACCTGCTGCAAGCCAACGGCGGCAAGGTTTGCGTGGC
GATTCAGAACCCGTTCACCATGGGCGACATCTGCAAGGGAGCCCTTGGCCAGTGGTAAGTCCTGAGGC
AGGGGTTTGCGAGGCCTATCCTTCTCAACTCAACTAAGATGACCTCCCGCGTACCCGCATCAATACCA
ATACAACGTCCATGGCCCCCCGGCTTTTACGGACACACCTGCACTTGTGTGCGGAACTTGTAACTTG
TAACTAACGAATGTACGGCCGCGCCACGTGCAAGAA**ACGCGT**TTTCCTTCCCGCACTTTTAGACCACA ***Mlu*I***
AGTTGCACACGTGTCAATTAGCAGCAGTGGTAGTAGGGATGAGTGTGCCTGATTGCTATGTCGCTAAT
AATACTCCTTCATGCCCCTGCTGTATCTTGCCTGCGCCCGTGCAGCTACGCCAGCATCTTCAACAGGG
ACAACTCGGACTACTGCTGCCCCATCTACCGCACGGGGCCA**TGA**TTGCCGTAAGAGCAGTCATGGCGC **stopp**
TGGAGAGCGCAGCAGCCGGAAGTAGGGGTAGAAGTTGTTGTTGCTACAAGTGCAGCAGCAGGCAGAAG
ATAAGGAGCACAATATGGCATCTTAGTACTGCATGAATGAACTGACATACAGACATGTTTACAGATCA
TCAGTCGGCGGGTTTTTCACTTTATCTCTTCGTTCGGTTGCAGCAGTCGATAGATAGATAGACAGACG
AGCTAGACAGTATGATTGATCTGTTGGACGGAATCTTTTCCTTTCCCCGACACAACTGCTTGTAGTAA
CATTTTTTGTAATGATTT**ATCGAT**GTACCACAATTCCTCTCGAACAGTTACGAGGCTAGATACTCGGA ***Cla*I***
CAGGCCTGGTAGGCAGGTGCTGCTGTGCTGCATATATCTATTAGGATGGTGGCGCAGAGGTTTCTTTG
GTGGGTTTGCATGTTGCCATAGACACCGCGGCCGGATGTGCACGGTTTATTAAATGATAATGTGGCTT
GTGAAATTAACGCAAGTAGCTGGCGAGCAGTGGCTACTTGGTCAAGTGGACTGAGTCTGAGCATGACG
TGGTGGGACGCGGTGCGGTCTATTTGGAGCACTAAAGTACATCTCGTCATCAGCAGGCTCTGCTGGGT
CCTGCGGGTGGCGCAAGTTTGAAACGAACGCGCGGTGGCACATTCTCGTGGGTGTCTTCACCAAGGAG
TTCCAAATCCTAAGAACAAGGTTAGCCCAATGGAACTGCAGTGGCGTCTGAGGTAGCTGACGGTAGAG
ACAGATCACGGTGATACAGTAACCTTTTACCGGGGCACATTGTGGCGACTGGAGCCGCATGACCGTCC
CCGTCACTTTGTAACCGCTCAAACTGCACGATAACTCCAACCCACACCGTCTTCAATGCGGCAGCTTG
CGACGGTTTCGC**AGATCT**TCCGAACTGCTCCAATAGCAGCTTGGGATCGACCCGTCATTTCTGCACCA ***Bgl*II**
AGTCGCAAAATAATGACAGATCCTAGGTGGCCGTGGTTATATGCTTTGCCACGTTGTAGAGTCCGAAC
TATATTGTCTTCCCTATGGGCACCCATGGCCATGCTGGATGGCGAAGCACAGTGGTGAGCGGCCTAAG

GTAGCTCTCCGACCAGGAAAACATCACCAGTGCACAGCTCGTCATAGCGATGAATGTTGCAGGGGTGC
AAAGGCATGTTCCACCACACCATTACGAGCAACCTTAGGACCGCGTTCCCCCAGGTAAACAGGGGAGG
TTTCAGGGGCTAGATATACTGGTCCAATAAGCAGGGTGCTATTGATGCAGGCGGTAATGTAGCACCCT
CCCTCATCCGACGTACCTCACTCCCTGCACATGTAGAGCCCCTGCGTAGAACGGCTATCTGTGATGAG
GGGGAGTCCCTGGTATAGAGGCCAATCATGCAAACTCGCTGTGCAGTGCTTCATCAGTTAGGGAGCAT
GTGCCGTTGTTGGACATTCGGGGACTGCGGGACAGCATTGTCAACATCGTTGCAGGCCCTGTAATGGG
CCACATCATGCAGTAGGTTTCTAGCGGTTTCCGAGGCCGAGGTCATTTTGCCGGGGTGTACGGCATTG
TTGCGGGTTGCGGGAAGTTGTACAGGTAAATGCACCGGCCACATTTACCAACATTGCTTTAAATATAA
GTAGACAACCGAGAAAGAACCAAAAGATGTCGGAAAGAATTTGGGCAGAGGCCGAAGCTCGCCTGGCA
AGCACTGCGCTGAACGGATAGAGAGAGACACGAATAAGAAGTGTACAACGGGGCCTCAATAGGCTTTG
GGGTCCATAGATACCGGGCGCCCTCGCTCTCAGGCATTAAGTCTCGGACGCGGGCCACAAGACCTGGA
GCTGCTTGGCTGTTCCCTCTCGTCCCCCCCCTTCAGAAGAAAAACTCTGACCACTATCCCTCTCTCCA
GTTCCCTCTCAGTCTGGGTGGTGCAATACCAGACCCCTCGCCACACACACCGCCACCTCAGCACCAAA
CCGTCACATTACGCTGCAAACATTGAGTCACGTTTGTTAGGCGCTAGCACGCTGTTGTATGAATTAGT
TTCTGAGTCTATGTTGATGTGGTTTGGTCCCGTTGCTGATGCAACTGGCTTTCGCATCATTAACCGCC
GAGTAGCAGCCGTAGCTACCGGTGCGGAAAACGATGCATGCTCACCAACCTAAATGATGAAGGGTGTG
TGGCGAAGTTATATTCATTATGCCAGGATCAGAGCTGTTGGTCAGTAAATATCAAGTTATATAAAAGA
AATGGTGCTACAGATTACAACGACGGGTCGGATTCCGACACGTACAACACGAGCAACATCCCAACGGA
TGAAATGCACATTTATATGTGTCTGCGATTAAC**GAATTC** ***Eco*RI**

Fig. S2. Genomic sequence of the *V. carteri phII* gene. The sequence depicted here corresponds to the 8329-bp genomic fragment utilized in plasmid pPhII-YFP. The *phII* gene (1) is located on scaffold 34 (nucleotides 980223 to 985045) of the *V. carteri* genome version 2.1 (2) in Phytozome v13 (3) on the reverse strand. The start codon is at nucleotide position 985025-985027 on the reverse strand. In the current *Volvox carteri* genome annotation available at Phytozome v13 (Volvox v2.1) pherophorin II is not annotated. Therefore, the gene structure was established based on older annotations and confirmed with RNA-Sequencing data (4). The gene structure is indicated as follows: Coding sequences are shown with blue background, UTRs with green background and the promoter region with grey background. Start and stop codons are highlighted (violet font). The 5' UTR is just 18 bp in length, while there is a quite long 3' UTR of 869 bp. The coding sequence totals 1557 nucleotides. The restriction sites that are shown in Figure 2 (main text) and SI Appendix, Fig. S12 A and B are marked (bold, underlined). Restriction sides that were used for inserting the *yfp* coding sequence are marked with asterisks.

GGATCCATGACTGGAAAACCCATCCATGACCAAGTCGTTCCCCGAGAGATAGCAGCTTTGGATCGACT *Bam*HI
CGTCGCTTGTGAAAGCCTACCTGGTTACTTAGACGGATTTAGCGACTAGACCACTTATGAAGGTGTTG
ATAAACACCGGGGGTTTCCCTTGGGGTTTTATAGTCGCTTGCAGTCCGTCTGAGGCGCTCGGGATAAT
ATGCCGTACAGTAGTAGATATTCCTGCAGAAGAGAGTCGCACCTCCAGGGAAGAGTTACAATACAGGT
CTGGCCGTGAACGGGCAGACGTGTACAAGATGGTGTACGGAATCGAGTATGTGCACGGATCGTTACAG
CCATGGCCGTAGCCATGTACTGCTGAGTACGGCTGTAGTCGAAAGATGACGAAGATAAGATAGTTTTA
TGTACTGTGTGTACGATATTCGCCTCGACGATGGAATGATAAGAAGGAATGGAGTCCCTACTCGGAAG
TCCTGAGCCAGGCTCTTACACTGGGCACACATTGCCATCCAATGGATGGCCATGTGAATCACAGTGGT
GCCCGGCCTAAGGTAGCTAAGGACTTGGCCGTTATCATCGGTAGATTGCTCACCCACCTTCTCCGACC
AGGAAAACATCCTCGTCACTGGTGCACAGCTCGTCATAGCGATGAATGTTGCAGGGGTGCAAAGGCAA
GTTCCACCACACCATTATGAGCAACCTTAGGACCGCGTTCC**CCCGGG**TAGACACAGGAGGTTTCAGGG *Sma*I
GCTAGATATACTGGTCCAATAAGCAGGGTGCTATTGATGTAGGCAGTAATGTAGCACCCTCCCTCATC
CGACGTACCTCACTCCCTGCACATGTAGAGCCCCTGCGTAGAACGGCTATCTGTGATGAGGGGGAGTC
CCTGGTATAGAGGCCAATGGGAGCATGTGCCGTTGTTGGGCATTCGAGGGGTGCGTGACAGCGTTGTC
ATCGGTAGGGTCGTGCCGAGTTGTGAAGACGGGCTCCTGTAATGGGCCTTATCATGCAGTAGGTTTCT
TGTGGTTTCCGAGGCCGAGGTCATTTTGCCGGGGTGTACGGCATTGTTGCGGGTTGCGGGAAGTTGTA
CAGGTAAATGCATCGGCCATTTTTATCAGCCTTGTTTAAATATAAGTAGACAACCGAGCAAGAACCAA
AGTTTGTCGGAAAGAATTTGGGCAGAGGCCGAAGCTCGCCTGGCAAGCACTGCGCTGAAGGGATAGAG
AGAGACACGAATAAGAAGTGTACAACGGGGCCTCAATAGGCTTTGGGGTCCAGATCGCTGACGCCCTC
GCTCTCAGGCATCAAGTCCCGGACATGGGC**CTTAAG**ACCTGGAGTTGCTGGGCTATTCCCTCTCGCCC *Afl*II
TCCCCGCTCTGACCACTATCCCTCTCTCCAGTTCCCTTTCAGTTCGGGTGGTGCAACGCCAGGCCCCT
CGCCACACACACCGCACCGCACCCACGTTTGTTAAGCGCTAGCAGGCTGTTG**TCTAGA**GCTGTAGAAG *Xba*I
TTCCTAGGTCTATGTTGATGTAGTTTGGTCCCGTTGCTGATGCAACTGGCTTTCGCATCATTAACCGC
CGAGTAGCAGCCGTAGCTACCGGTGCGGAAAGGATGCATGCTCACCAACCGAAATGATGAAGTGCGTG
TGGCGAAGTTATATTCATTATGCCAGGATCAGAGATGTTGGTCAGTAAATAGCAAGTTATATAAAAGG
AATGGTGCTACAGATTACAACGACGGGTCGGATTCCGAAACGTACAACACGAGCAACATCCCAACGGA
TGAAATGCACATTTATATGTGTCCGCGACTAAC**GAATTC**TGCGCTGTGTTTCTAAGATTTATATTCTG *Eco*RI
CATAAAGATATTGATTCCAAAGGACGAAGCGCCTCTGTTGCGGTGGCCAGTCTCGGAAGCGGCTACCT
GCGCCCGCCGTTTACGGTAGTCGTACGGCCCCGCAGCAGCACAGCTGCCGCGGTCGGTCGGGTAGATA
AATAGCGCCACTATGATACAGAACATCATCAAAGTGACATAGCTCTGACAAT**ATG**GCATACTTTGCAA start
AGGTAGCTTTCATTTTGGCGACAGCGCTGGCGGTTGGCTGGTGCGTTTTTAGCATGGTGCATTCCCAT
ATGCCTGGGCTTACAATCTGAGGTTGGGTCTATATATTTAAGTATATTTAAGAGCCATATACTTGAAG
GCTTTCAGTCTTTGCCTTTGCGTCGCAAAAGACGTACATGTCCTGTCGCATGCCATAGTGATGAGCCT
TTTCTATAAAGCAATACATAAAAGATAAGACGTCTACAATCATAACTCGATCATGAAACAGCAAGCGT
CAACCATGAGCACTGACCTTGCTTGCTGCTTGTCGCCGCTGGTTCCAGGGCGCTCAGCGCTCATGCGC
AGGAATACAACGAGTACGATCCCTCAGCTGTCACGCCTGGCAGCATCCCCAACTTCCCATTCCGCGAC
TGCAACACCACAAACGGCGCGTACCGGCTGGCACCGGTGTGGCGGCCTTCGGGCAGCAACACGTACTG
CTTCAAGATCCAGGTCAGCCAGGATGCCCTGTCCTGCACTGGCGCCTGCTGCAGCGCCGACTTGCACA
AAATTGAGGTGAGCCCACGGCCGGCTGATGGTGTGGGGGATGCGACATGCGGACACAGTGCTACAGGT
CCTAGGGCCTCGGAAAGCACGGGAAGAGGCTCATTGGGCCCGCAACCAAATGTCAACGATTATTTTCC
AGTAGTTTCTCATGTGTGTGTGTGTGTGCATGTGCGCATGATGTCGGTGTTGAGGGCTCCGTTACCAT
CCTAGCTGCTCCTCCGGGGAAGTGGATTTCTAATCAGGGTTCGGAACGCGCCTCACAATATTTACATA
GCTCATTTACATGTGCTGGAGAGAATGAGAAGTTCGCACCCTCAAAAGTAGAGGGAACATCAAATTGT
GATGTTCCCTCCTGCCTCACCTTGCCCTTACTCCATCCTCCCACCACATGCCACCCCGCCATGTCCGC
AGTTCAACGTGTCCAGCAGCTGCCTGGTGGCCGGGGCCTCAGTTGTCGCCACCGTGAATGGAGTGCGG
A**CCCGGG**TTGGTGCGACCCTGGACAAGGCCCCGGCTGGGCCTGTTGGCTCCGCCGTTCTGCGGCTCAC *Sma*I
CCAGCTGGGGCTGGACACCACGACCGCCCAGGATGCGGAGGTGTGCCTCACCCTCAAGACCAACCGCG
GAGGCCAGGGCTGCACCACCCTGGAGCAACTGTGCTCGTCGCCAGGCTTCCCTGCAGGGACATGCACA
GCAGCTATGTTCGATGTCGCGTGTGATTGCTGCCCGGTTTCTCAAGCCGGTCAGGCCCTCCCGCCGCC
GCCACCAGTTATTCCGCCGGTCTTCCGGCCTTGTGACGTCTGCATCGCCGCGACCATTGTGCCCCCGG
CCAATGATGTGCGACCATACCGCTACGACAGCGCCACCTGCGCCGCCATTCAGCAGAACATCGCCGAT

GCCATGAACTCTCTCCTGGGAGGCGCCAACATCGGCGCCTTCTCACCTTTCGCGCCAAATGCAAGCCA
GTGCTTTGACACACAAATCATTACATGTGGCAGGTTCAACGGTTCGGACAGTGATGCGTTGGCCAATC
TGACGGAAGCAGTGCAGCAGCAGCTTTCCGCCTTCATTGGAGTCGCGTCTGGCGGCAACGTCTGCAAT
CCAAAGTTGGAGAAATACACGGTGACGGTCTCCACCATCAACAATGTAGCAGATCAGTGCCTGGACCT
CTCGCAGTCGGCTAGTTGCTTCCTGCCTGGCGTTCCATTCCCCAACTGCACGTGAGTCACCCTACTTC
ATGGCGTCGTCTCTTTTGCGGCGCGGCCGCCCTCCGTAAAGATTTGGGGGCGAGGATGTAGGGCCTAA
TAACCAAAGCTTCCCCCCGCAATGGATGGACCCATGTTTGGGATCTGGCTAGCGTCTTTGTCGTGTAT
GCAGAGCTTTGGATGAATGGCTGAAGGCAAAGACACCGCGCTTGCATGGATACCTTGCTCACATGGCT
ATTTGAAAAAATCAGGAACGTTGGGTGAATATGTGATAGTACTAGTTACCGACCCTGCTGCCGCCTCC
GCTCGCCCTCTGACGCGCAGGTGCAACACAACCCAGGGAGTCATGCCGTTCACAGTCTCCCCCACCTG
GTATGCACAGCCGGCCAATGTGCGCTGGGGCCGCAACGTCACTGAGTACTGCTTCACCGTCAACACGC
TACAGCCAAGTCAGGTCGTGCCGGTGAGGCCAGATAGGTGCATGGAGGACCCGTGGGGTGGCGCTTTG
TTTTCTTCCACGCACCCATGCATGCTTGGCTGCATGCCTGATGAGCTCGGTTAGTTGTATCGGGTCTG
GACAAGGCACACGGGCCTGTCTGCTGCGCGTAGCTCACCCAGACAAAGTTAAGAAATTACATGACTGA
GGATGCTGCCTCGTTCGCTGTCCCCCTGCAGAGCACCTGCTACAACGCAAACGACGCGCTCGCCAAGA
TCGAGTGGTATGCAAGTAAGTTATCATTGCTGTTGCTTGCTTGCGTAGTCTTCTATCGTTTCAACGTT
TCTTAGATAAGACCCGTCCTTTCAACCTGGGCAGACATGCATGGATATGTGGGAGTACGGCATGCGAA
TGGATATGGATATGTGCAAACTTCTACTGTCGCAGCATGATGTGAACTCTGGCCCTGCCGGCTGCACA
CATACGCATACATGACTTCCGTAATGCGAGCAGACTGCAGCATAGCACCCAGTACGGCAGCATCAGTG
GTTAACCTTTTTTGGTCCTCGCTACAGCCTAGGGTTTCACCTGCCGTGCATTTTGGCTTGTGTTTCCA
TGACTGCAGGTGATGCGTTCAGGTCGGCGGTCAAGGGTTTCACGGTGTACCCCGCCGGGGGATCAAAC
AAGACCATTGCCGACAGCTGGGGCGCCACTGGAACCGATACCCTGAAAGGTGAGGCCGGCAAGCTAGA
TAGCAGCTCAGGTAGATGCACCTCGTGCGGGGCTCATTCGCTGCTTCGATTGTGGATTCTGTGCGGCA
CTTACCTGCCGTGTGTCACAAGGGTAACTGTCGTGCGAGACCTTCACTTACAATACACTCGAGGAAAT
CTCGACGGACCTGCATCTCGGAAGGCCTCTTCGATGACCCTATCAAAAGATCAGCAATAAGCTCCAAC
AGTGTATGAAGATTGACGGAGCTGTCAAGCTCAATAACATAATCCCGAAAATGGCAAGCACTACGGCT
ACTGTATAAAACCCAGCGCAGAGGCTACACTCCCTTCGCTCCTGGCGCATCTAAGCTGCAATTACAAT
TACAGTAACTCTGTTCAGTAGCCAGCCATGCGCCGGTTTGTTTGTACCGTACCTGTCCAATTGCCATG
TGCTGCACCATCACTGCTAATGCCTGCATGTCAAATAAGATCACATCGTTCCCTGCCTGCGCATCCTT
TTGTTCGTGTGTGCAGTCAATCTCAACTGGAACCTGCTGCAAGCCAACGGCGGCAAGGTTTGCGTGGC
GATTCAGAACCCGTTCACCATGGGCGACATCTGCAAGGGAGCCCTTGGCCAGTGGTAAGTCCTGAGGC
AGGGGTTTGCGAGGCCTATCCTTCTCAACTCAACTAAGATGACCTCCCGCGTACCCGCATCAATACCA
ATACAACGTCCATGGCCCCCCCGGCTTTTACGGACACACCTGCACTTGTGTGCGGAACTTGTAACTTG
TAACTAACGAATGTACGGCCGCGCCACGTGCAAGAA**ACGCGT**TTTCCTTCCCGCACTTTTAGACCACA ***MluI****
AGTTGCACACGTGTCAATTAGCAGCAGTGGTAGTAGGGATGAGTGTGCCTGATTGCTATGTCGCTAAT
AATACTCCTTCATGCCCCTGCTGTATCTTGCCTGCGCCCGTGCAGCTACGCCAGCATCTTCAACAGGG
ACAACTCGGACTACTGCTGCCCCATCTACCGCACGGGGCCA**GGTACC**GGCGGAGGCGGTGGCATGAGC ***Kpn*I**
AAGGGCGAGGAGCTGTTCACCGGCGTGGTGCCCATCCTGGTGGAGCTGGACGGCGACGTGAACGGCCA 5xG spacer
CAAGTTCAGCGTGAGCGGCGAGGGCGAGGGCGACGCCACCTACGGCAAGCTGACCCTGAAGCTGATCT
GCACCACCGGCAAGCTGCCCGTGCCCTGGCCCACCCTGGTGACCACCCTGGGCTACGGCCTGCAGTGC
TTCGCCCGCTACCCCGACCACATGAAGCAGCACGACTTCTTCAAGAGCGCCATGCCCGAGGGCTACGT mVenus coding sequence
GCAGGAGCGCACCATCTTCTTCAAGGACGACGGTAACTACAAGACCCGCGCCGAGGTGAAGTTCGAGG
GCGACACCCTGGTGAACCGCATCGAGCTGAAGGGCATCGACTTCAAGGAGGACGGCAACATCCTGGGC
CACAAGCTGGAGTACAACTACAACAGCCACAACGTGTACATCACCGCCGACAAGCAGAAGAACGGCAT
CAAGGCCAACTTCAAGATCCGCCACAACATCGAGGACGGCGGCGTGCAGCTGGCCGACCACTACCAGC
AGAACACCCCCATCGGCGACGGCCCCGTGCTGCTGCCCGACAACCACTACCTGAGCTACCAGAGCAAG
CTGAGCAAGGACCCCAACGAGAAGCGCGACCACATGGTGCTGCTGGAGTTCGTGACCGCCGCCGGCAT
CACCCTGGGCATGGACGAGCTGTACAAG**GGTACC**TGATTGCCGTAAGAGCAGTCATGGCGCTGGAGAG ***Kpn*I, stopp**
CGCAGCAGCCGGAAGTAGGGGTAGAAGTTGTTGTTGCTACAAGTGCAGCAGCAGGCAGAAGATAAGGA
GCACAATATGGCATCTTAGTACTGCATGAATGAACTGACATACAGACATGTTTACAGATCATCAGTCG
GCGGGTTTTTCACTTTATCTCTTCGTTCGGTTGCAGCAGTCGATAGATAGATAGACAGACGAGCTAGA
CAGTATGATTGATCTGTTGGACGGAATCTTTTCCTTTCCCCGACACAACTGCTTGTAGTAACATTTTT
TGTAATGATTT**ATCGAT**GTACCACAATTCCTCTCGAACAGTTACGAGGCTAGATACTCGGACAGGCCT ***ClaI****

GGTAGGCAGGTGCTGCTGTGCTGCATATATCTATTAGGATGGTGGCGCAGAGGTTTCTTTGGTGGGTT
TGCATGTTGCCATAGACACCGCGGCCGGATGTGCACGGTTTATTAAATGATAATGTGGCTTGTGAAAT
TAACGCAAGTAGCTGGCGAGCAGTGGCTACTTGGTCAAGTGGACTGAGTCTGAGCATGACGTGGTGGG
ACGCGGTGCGGTCTATTTGGAGCACTAAAGTACATCTCGTCATCAGCAGGCTCTGCTGGGTCCTGCGG
GTGGCGCAAGTTTGAAACGAACGCGCGGTGGCACATTCTCGTGGGTGTCTTCACCAAGGAGTTCCAAA
TCCTAAGAACAAGGTTAGCCCAATGGAACTGCAGTGGCGTCTGAGGTAGCTGACGGTAGAGACAGATC
ACGGTGATACAGTAACCTTTTACCGGGGCACATTGTGGCGACTGGAGCCGCATGACCGTCCCCGTCAC
TTTGTAACCGCTCAAACTGCACGATAACTCCAACCCACACCGTCTTCAATGCGGCAGCTTGCGACGGT
TTCGC**AGATCT**TCCGAACTGCTCCAATAGCAGCTTGGGATCGACCCGTCATTTCTGCACCAAGTCGCA ***Bgl*II**
AAATAATGACAGATCCTAGGTGGCCGTGGTTATATGCTTTGCCACGTTGTAGAGTCCGAACTATATTG
TCTTCCCTATGGGCACCCATGGCCATGCTGGATGGCGAAGCACAGTGGTGAGCGGCCTAAGGTAGCTC
TCCGACCAGGAAAACATCACCAGTGCACAGCTCGTCATAGCGATGAATGTTGCAGGGGTGCAAAGGCA
TGTTCCACCACACCATTACGAGCAACCTTAGGACCGCGTTCCCCCAGGTAAACAGGGGAGGTTTCAGG
GGCTAGATATACTGGTCCAATAAGCAGGGTGCTATTGATGCAGGCGGTAATGTAGCACCCTCCCTCAT
CCGACGTACCTCACTCCCTGCACATGTAGAGCCCCTGCGTAGAACGGCTATCTGTGATGAGGGGGAGT
CCCTGGTATAGAGGCCAATCATGCAAACTCGCTGTGCAGTGCTTCATCAGTTAGGGAGCATGTGCCGT
TGTTGGACATTCGGGGACTGCGGGACAGCATTGTCAACATCGTTGCAGGCCCTGTAATGGGCCACATC
ATGCAGTAGGTTTCTAGCGGTTTCCGAGGCCGAGGTCATTTTGCCGGGGTGTACGGCATTGTTGCGGG
TTGCGGGAAGTTGTACAGGTAAATGCACCGGCCACATTTACCAACATTGCTTTAAATATAAGTAGACA
ACCGAGAAAGAACCAAAAGATGTCGGAAAGAATTTGGGCAGAGGCCGAAGCTCGCCTGGCAAGCACTG
CGCTGAACGGATAGAGAGAGACACGAATAAGAAGTGTACAACGGGGCCTCAATAGGCTTTGGGGTCCA
TAGATACCGGGCGCCCTCGCTCTCAGGCATTAAGTCTCGGACGCGGGCCACAAGACCTGGAGCTGCTT
GGCTGTTCCCTCTCGTCCCCCCCCTTCAGAAGAAAAACTCTGACCACTATCCCTCTCTCCAGTTCCCT
CTCAGTCTGGGTGGTGCAATACCAGACCCCTCGCCACACACACCGCCACCTCAGCACCAAACCGTCAC
ATTACGCTGCAAACATTGAGTCACGTTTGTTAGGCGCTAGCACGCTGTTGTATGAATTAGTTTCTGAG
TCTATGTTGATGTGGTTTGGTCCCGTTGCTGATGCAACTGGCTTTCGCATCATTAACCGCCGAGTAGC
AGCCGTAGCTACCGGTGCGGAAAACGATGCATGCTCACCAACCTAAATGATGAAGGGTGTGTGGCGAA
GTTATATTCATTATGCCAGGATCAGAGCTGTTGGTCAGTAAATATCAAGTTATATAAAAGAAATGGTG
CTACAGATTACAACGACGGGTCGGATTCCGACACGTACAACACGAGCAACATCCCAACGGATGAAATG
CACATTTATATGTGTCTGCGATTAAC**GAATTC** ***Eco*RI**

Fig. S3. Genomic sequence of the *V. carteri phII* gene with *yfp* coding sequence added. The sequence depicted here is present in plasmid pPhII-YFP. The chimeric gene consists of 8.3 kb genomic DNA including 5’ and 3’ regulatory sequences and the complete transcribed region of pherophorin II including all introns as present on scaffold 34. The *yfp* (mVenus) (8) coding sequence was fused in frame with the coding sequence of the last *phII* exon and a 15 bp sequence coding for a pentaglycine flexible spacer. For the insertion of mVenus, artificial *Kpn*I sides were introduced by recombinant PCR which also allow for a later exchange of the fluorescent marker. The gene structure is indicated as follows: Coding sequences are shown with blue background, UTRs with green background and the promoter region with grey background. Start and stop codons are highlighted (violet font). The 5’ UTR is just 14 bp in length, while there is a quite long 3’ UTR of 1,168 bp. The coding sequence of mVenus is highlighted in yellow. The pentaglycine spacer is highlighted in orange. The restriction sites that are shown in SI Appendix, Fig. S12 A and B are marked (bold, underlined). Restriction sides that were used for inserting the *yfp* coding sequence are marked with asterisks.

2. Supplementary methods: semi-automated image segmentation and geometric analysis

A. Overview. We employ a semi-automated image analysis pipeline which uses Cellpose (9) as a key step.

1. Contrast stretching of the image is performed by predetermined cutoffs, e.g. 2nd and 98th percentile intensity.
2. A J-invariant filtration is performed using (depending on the channel, fluorescence or trans-PMT) either (i) total-variation (TV) denoising by minimizing the Rudin-Osher-Fatemi functional
$$\min_{u\in \mathrm{BV}(\Omega)} \int_\Omega \left[\|\nabla u\| + \frac{\lambda}{2}(f-u)^2\right] \qquad [1]$$
where f is the intensity profile of an image supported on the image domain Ω to be denoised, or (ii) wavelet denoising with adaptive thresholding. A J-invariant filter is defined as one whose output value at every pixel is independent of the value of the source pixel (i.e. is only a function of source pixels at other locations). We find TV denoising in particular to be effective at filtering Poisson noise and significantly enhance the performance of Cellpose on low-SNR fluorescence images. We did not use the trained denoising model available in Cellpose3 (10).
3. A user-prompted input polygon P is used to estimate the diameter $d = \max\{\|\boldsymbol{v}_i - \boldsymbol{v}_j\| \mid \boldsymbol{v}_i, \boldsymbol{v}_j \in P\}$ of typical instances to be identified in the image, passed as the *diameter* input to the Cellpose *cyto3* model (10).
4. Objects identified as pixel-space masks by Cellpose are converted to polygons in the plane by either (i) taking the convex hull, for convex objects such as somatic cells, or (ii) identifying outlines in the mask. Degenerate and invalid polygons are suppressed by taking a single binary erosion-dilation step. Further conversion to ellipses for approximately elliptical objects (such as parent and offspring spheroids) is performed by computing the ellipse with the same nth-order moments of area as the polygon (see §B) up to $n = 2$.
5. False-positives are manually rejected where identified. False-negatives, where identified, are re-prompted to Cellpose by restricting to a user-specified region of interest around the object, and re-iterating from step 1.
6. For identification of the somatic CZ3 geometry in particular, only the somatic cell-CZ3 compartment pairs (as seen in Fig. 5, main text) which have jointly been successfully identified are retained.
7. Downstream analysis of the resulting polygons and/or ellipses is performed as described in Table 1 (main text) and further detailed below in §B.

B. Geometric moments of area. The geometric moments of bounded planar domains, analogous to the moments of bivariate uniform random variables, quantify shape properties such as size, center of mass, eccentricity, skew, and so on. In the case of polygonal domains, the geometric moments up to sufficiently high order completely determine the vertices (11).

Definition 2.1 (Planar nth area moment tensor)**.** Let $D \subset \mathbb{R}^2$ be an open measurable set with boundary given by a simple closed curve $\partial D = C$. The nth moment tensor for the domain D of uniform mass density is
$$\mu_I^{(n)}(D) = \iint_D \boldsymbol{x}^I d^2\boldsymbol{x} \qquad [2]$$
where $I = (i_1, \ldots, i_n)$ is a multi-index such that $i_j \in \{1, 2\}$ and
$$\boldsymbol{x}^I = \prod_{j=1}^n x_{i_j}. \qquad [3]$$

The 0th moment $\mu^{(0)}$ is the area of D. Accordingly, one may define the radius R_D of an equivalent (same-area) circle as $R_D = \sqrt{\mu^{(0)}/\pi}$. The nth-order moments can be calculated for polygons by (i) applying the divergence theorem to write Eq. (2) as a boundary integral on C, and (ii) computing the integral as a finite sum using the piecewise-linearity of the sides C.

B.1. First moment and centrality.

Definition 2.2 (Centroid)**.** The center of mass of D is
$$\mu_j = \frac{\mu_j^{(1)}}{\mu^{(0)}}. \qquad [4]$$

The notation $\boldsymbol{\mu} = [\mu_1, \mu_2]$ evokes the probabilistic interpretation as the expected value of a uniform distribution supported on D. The basis in which Eq. (2) is computed will unless otherwise specified be taken, for moments of order ≥ 2, to be one in which $\boldsymbol{\mu}$ is at the origin.

Definition 2.3 (Dimensionless centrality of a test point)**.** For a test point $\boldsymbol{y} \in \mathbb{R}^2$, we define the centrality metric

$$d_{\boldsymbol{\mu}}(\boldsymbol{y}) = \|\mathsf{W}(\boldsymbol{y} - \boldsymbol{\mu})\| = \sqrt{(\boldsymbol{y} - \boldsymbol{\mu}) \cdot \Sigma^{-1}(\boldsymbol{y} - \boldsymbol{\mu})} \quad [5]$$

with W a matrix defined as $\mathsf{W}^\intercal \mathsf{W} = \Sigma^{-1}$, and Σ the covariance matrix of D, defined in Eq. (7).

This *whitening* procedure (again evoking the probabilistic interpretation of Σ) enables comparison across domains D of varying second moment, resulting in a quantity which is dimensionless. In probability terms, Eq. (5) is the Mahalanobis distance of $\boldsymbol{y}$ to the uniform distribution supported on D.

In particular, d is scale-invariant; for dilations of space $\mathbb{R}^2 \mapsto \rho\mathbb{R}^2$, $\rho > 0$, we have $\boldsymbol{\mu} \mapsto \rho\boldsymbol{\mu}, \boldsymbol{y} \mapsto \rho\boldsymbol{y}$, and $\Sigma \mapsto \rho^2\Sigma$ by Eq. (7), hence $d_{\boldsymbol{\mu}}(\boldsymbol{y}) \mapsto d_{\boldsymbol{\mu}}(\boldsymbol{y})$ by Eq. (5). Moreover, dilations preserve aspect ratios, since

$$\Sigma \overset{\mathbb{R}^2 \mapsto \rho\mathbb{R}^2}{\mapsto} \frac{1}{\rho^2 |D|} \iint_D \rho(\boldsymbol{x} - \boldsymbol{x}') \otimes \rho(\boldsymbol{x} - \boldsymbol{x}')\rho^2 d^2\boldsymbol{x}' = \rho^2\Sigma, \quad [6]$$

hence its eigenvalues map as $\lambda_j \mapsto \rho^2\lambda_j$ and the ratio $\lambda_{\max}/\lambda_{\min}$ is preserved.

B.2. Second moment and isotropy.

Definition 2.4 (Covariance matrix of a domain)**.** The normalized second central moment, or covariance matrix, is

$$\Sigma = \frac{\mu^{(2)}}{\mu^{(0)}} \quad [7]$$

As before, *central* indicates that $\mu^{(2)}$ is computed in a basis in which $\boldsymbol{\mu}$ is at the origin.

In probability terms, Σ is the covariance matrix of a uniform distribution supported on the domain D. If D has radial symmetry about $\boldsymbol{\mu}$ (either continuous, as a circle does, or discrete, as regular n-gons do), then $\Sigma = c\mathsf{I}$ for some $c > 0$. This is verified by the existence of an eigenspace of dimension 2. $\Sigma^{-1} = \mathsf{M}$ is the matrix defining an ellipse with the same aspect ratio as D.

Definition 2.5 (Principal axes of a domain)**.** Using Eq. (7), we may define the principal axes and stretches of a domain D via Hermitian eigendecomposition

$$\Sigma = \mathsf{P}\Lambda\mathsf{P}^{-1} \quad [8]$$

with $\boldsymbol{v}_i$ the columns of P being the principal axes and and $\lambda_i > 0$ in ascending order (as Σ is symmetric positive-definite for non-degenerate curves).

Definition 2.6 (Aspect ratio of a domain)**.** Let λ_1, λ_2 and v_1, v_2 be the principal stretches and axes (in ascending order) of Σ as in Eq. (8). The aspect ratio is

$$\alpha = \sqrt{\frac{\lambda_2}{\lambda_1}}. \quad [9]$$

Ellipses (objects defined uniquely by their moments to order $n = 2$) with the same orientation, aspect ratio, and area (πab) as D have the minor and major axes

$$a = \sqrt{\frac{\mu^{(0)}}{\pi\alpha}}, \quad b = \alpha a. \quad [10]$$

Eq. (10) is an exact elliptical representation of a polygon (or arbitrary domain), in contrast to elliptical approximations, e.g. (i) weighted ℓ^2-minimization of vertex distance or (ii) convex programs for minimum-area bounding ellipses.

B.3. Moments under affine transforms. Let $\mathsf{F} > 0$ be a symmetric positive-definite matrix (e.g. a strain tensor) and define an affine transform of a domain D about its center of mass by

$$T(\boldsymbol{x}) = \mathsf{F}(\boldsymbol{x} - \boldsymbol{\mu}) + \boldsymbol{\mu}. \quad [11]$$

Let $T(D)$ be the transformed region. By change of coordinates for integrals, the relevant moments of $T(D)$ are

$$\mu^{(0)}(T(D)) = \iint_{T(D)} d^2\boldsymbol{y} = \iint_D \det\mathsf{F} d^2\boldsymbol{x} = \mu^{(0)}(D)\det\mathsf{F} \quad [12]$$

$$\mu^{(1)}(T(D)) = \iint_{T(D)} y d^2\boldsymbol{y} = \iint_D (\mathsf{F}(\boldsymbol{x} - \boldsymbol{\mu}) + \boldsymbol{\mu})\det\mathsf{F} d^2\boldsymbol{x} = \mu^{(1)}(D)\det\mathsf{F} \quad [13]$$

$$\boldsymbol{\mu}(T(D)) = \mu^{(1)}(T(D))/\mu^{(0)}(T(D)) = \boldsymbol{\mu}(D) \quad [14]$$

$$\mu^{(2)}(T(D)) = \iint_{T(D)} (\boldsymbol{y} - \boldsymbol{\mu}) \otimes (\boldsymbol{y} - \boldsymbol{\mu})d^2\boldsymbol{y} = \iint_D \mathsf{F}(\boldsymbol{x} - \boldsymbol{\mu}) \otimes \mathsf{F}(\boldsymbol{x} - \boldsymbol{\mu})\det\mathsf{F} d^2\boldsymbol{x} = \mathsf{F}\mu^{(2)}(D)\mathsf{F}^\intercal\det\mathsf{F} \quad [15]$$

$$\Sigma_{T(D)} = \mu^{(2)}(T(D))/\mu^{(0)}(T(D)) = \mathsf{F}\Sigma_D\mathsf{F}^\intercal \quad [16]$$

hence the centroid is preserved and the covariance matrix maps as $\Sigma \mapsto \mathsf{F}\Sigma\mathsf{F}^\intercal$.

B.4. Whitening a domain. Let a domain D have covariance matrix Σ Eq. (7). Define the *whitened* domain D_W by the affine transform T as defined in Eq. (11),

$$D_W = \{T(\boldsymbol{x}) \mid \boldsymbol{x} \in D\}, \quad \mathsf{F} = \Sigma^{-1/2}, \tag{17}$$

with the matrix square root F typically approximated by singular value decomposition (SVD) as

$$\mathsf{F} \overset{\varepsilon \to 0^+}{=} \mathsf{U}(\mathsf{S}+\varepsilon)^{-1/2}\mathsf{U}^*, \quad \Sigma = \mathsf{U}\mathsf{S}\mathsf{V}^\intercal, \tag{18}$$

and F the *ZCA whitening matrix* (12) with regularization constant $\varepsilon \ll 1$. Then D_W has aspect ratio 1 as defined in Eq. (9).

C. Isoperimetric problems. We recall here several quantities which can be used as measures of the deviation of a domain D from a disk.

C.1. Classical isoperimetric inequalities. One has

$$4\pi A \leq L^2, \tag{19}$$

where A is the area of D and L the total arclength of C (which we may now require to be a rectifiable Jordan curve) and is an equality only for circles. Accordingly, one defines an *isoperimetric quotient*, which we term the *circularity* in the main text,

$$q = \frac{\sqrt{4\pi A}}{L} \in [0,1], \tag{20}$$

maximized for disks. For regular n-gons, one has

$$q_n = \sqrt{\frac{\pi}{n}\cot\frac{\pi}{n}}. \tag{21}$$

A natural question is whether Eq. (19) can be used to define a set distance, in the sense of Hausdorff metric, of D to the "best" disk. This turns out (13) to be related to the problem of making Eq. (19) *quantitative*, in the sense of a nonnegative quantity $\nu(D)$ such that

$$4\pi A + \nu(D) \leq L^2 \tag{22}$$

for all D, with $\nu(D) = 0$ iff D is a disk. Without proof, we cite (13) the result that the *isoperimetric deficit*, defined as the dimensionless quantity

$$\mathrm{ID}(D) = \frac{1-q}{q} \tag{23}$$

upper-bounds any such quantitative inequality $\nu(D)$ via

$$\nu(D) \leq C_2\sqrt{\mathrm{ID}(D)} \tag{24}$$

for some dimension-dependent constant C_2. For convex D (applicable e.g. to the cells of a Voronoi tessellation), Eq. (23) upper-bounds the Hausdorff distance d_H to the best-fit equal-volume ball B as

$$\inf_{\boldsymbol{x}\in\mathbb{R}^2} d_H(D, B+\boldsymbol{x}) \leq C_2 \mathrm{ID}(D)^{\alpha_2} \tag{25}$$

for dimension-dependent constants C_2, α_2 (13). Equality is achieved for D which is a ball.

C.2. Weighted isoperimetric inequalities. Recalling the second moment M_2 as defined in the main text, eq. 1, written here with explicit arguments $M_2(\boldsymbol{x}, D)$, we recall by classical results that its minimization is also an isoperimetric problem.

Lemma 1 (Disks minimize $\mathrm{Tr}(M_2)$)**.** Let $D \subset \mathbb{R}^2$ be as in Definition 2.1, $|D|$ its Lebesgue measure, and $\boldsymbol{x} \in \mathbb{R}^2$. We have $\mathrm{Tr}(M_2(\boldsymbol{x}, D)) \geq \frac{|D|^2}{2\pi}$ with equality iff D is a disk and $\boldsymbol{x}$ is its centroid.

Proof. Write $\mathrm{Tr}(M_2)$ as

$$U(\boldsymbol{x}, D) = \mathrm{Tr}(M_2(\boldsymbol{x}, D)) = \iint_D \mathrm{Tr}[(\boldsymbol{x}'-\boldsymbol{x})\otimes(\boldsymbol{x}'-\boldsymbol{x})]\mathrm{d}^2\boldsymbol{x}' = \iint_D \left\|\boldsymbol{x}'-\boldsymbol{x}\right\|^2 d^2\boldsymbol{x}'. \tag{26}$$

Then optimality in the first argument implies

$$0 = \frac{\partial U}{\partial \boldsymbol{x}} = 2\iint_D (\boldsymbol{x}'-\boldsymbol{x})d^2\boldsymbol{x}', \tag{27}$$

which holds iff $\boldsymbol{x} = \boldsymbol{\mu}$ is the centroid of D. Locating $\boldsymbol{\mu}$ at the origin without loss of generality, we have

$$U(\boldsymbol{x}, D) \geq U(\boldsymbol{\mu}, D) = \iint\limits_D \left|\boldsymbol{x'}\right|^2 d^2\boldsymbol{x'}. \qquad [28]$$

Recognizing the last expression as the polar moment of inertia and applying a *weighted* isoperimetric inequality (7.2, (14)),

$$\geq \iint\limits_{B_{R_D}(0)} \left|\boldsymbol{x'}\right|^2 d^2\boldsymbol{x'}, \qquad [29]$$

where $B_{R_D}(0)$ is a disk at the origin of the same area as D (i.e. R_D is D's circular radius as in §B). Thus

$$= \frac{\pi R_D^4}{2} = \frac{|D|^2}{2\pi}. \qquad [30]$$

□

Recall also the (standardized) sum of second moments, Eq. 3, main text. Using the previous lemma we may establish the following optimality for it in terms of honeycombs.

Lemma 2 (Second moment bound for tessellations)**.** For any space packing $\{\boldsymbol{x}_i\}_{i=1}^n, \{D_i\}_{i=1}^n$ with standardized sum of second moments m_2 defined as

$$m_2 = n \sum_{i=1}^n \mathrm{Tr}(\mathsf{M}_2^{(i)}(\boldsymbol{x}_i, D)) / \left(\sum_{i=1}^n |D_i|\right)^2, \qquad [31]$$

we have $m_2 \geq \frac{1}{2\pi}$.

Proof. Let $A = \sum_{i=1}^n |D_i|$ and $\mathsf{M}_2(\boldsymbol{x}_i, D_i) = \mathsf{M}_2^{(i)}$. Then

$$m_2 = \frac{n}{A^2} \sum_{i=1}^n \mathrm{Tr}\,\mathsf{M}_2^{(i)} \qquad [32]$$

$$\overset{\mathrm{CS}}{\geq} \frac{1}{A^2} \left(\sum_{i=1}^n (\mathrm{Tr}\,\mathsf{M}_2^{(i)})^{1/2}\right)^2 \qquad [33]$$

$$\overset{\mathrm{L1}}{\geq} \frac{1}{A^2} \left(\sum_{i=1}^n \frac{|D_i|}{\sqrt{2\pi}}\right)^2 \qquad [34]$$

$$= \frac{1}{2\pi}, \qquad [35]$$

where the first inequality is Cauchy-Schwarz and the second is Lemma 1. □

Note that the first inequality is sharp only for configurations consisting of congruent cells D_i. The second inequality cannot be sharp for space *partitions*, only *packings* consisting of congruent disks. Space *partitions* optimize the second only if they are asymptotically (in n) regular hexagonal lattices (15, 16).

D. Average number of neighbors in a spherical Voronoi tessellation. Let $\{\boldsymbol{x}_i\}_{i=1}^n$ be a collection of points on the surface of a sphere and $\{V_i\}_{i=1}^n$ the corresponding set of spherical polygons given by their Voronoi tessellation. The number of neighbors of each V_i is precisely the degree (number of incident edges) $d(\boldsymbol{x}_i)$ of each node in the topological dual, the Delaunay triangulation $\{T_j\}_{j=1}^f$ of $\{\boldsymbol{x}_i\}$. Let m be the number of edges; since this is a triangulation of a compact (boundaryless) surface, we have the relation $3f = 2m$. By the Euler theorem,

$$n - m + f = \chi = 2 \qquad [36]$$

where χ is the Euler characteristic of the sphere. Substituting the triangulation property into Eq. (36) yields $m = 3n - 6$, hence the average degree $\overline{d}$ is

$$\overline{d} = \frac{1}{n} \sum_{i=1}^n d(\boldsymbol{x}_i) = \frac{2m}{n} = \frac{6n - 12}{n} \overset{n\to\infty}{\longrightarrow} 6, \qquad [37]$$

thus the average number of neighbors of a Voronoi polygon is asymptotically 6.

E. Distortion correction of compartment features due to elastic deformation against a glass slide. Segmented compartment features as described in §2.A occur in a single or tight range of focal planes due to the physical planarity of the surface, which arises from the mounting procedure described in Materials and Methods in the main text. This can induce some elastic distortion of the geometry of the compartments, as the surface of the organism itself is generally ellipsoidal and cannot be embedded isometrically in the plane. This distortion varies from spheroid to spheroid and also in principle depends on the constitutive (stress-strain) relations of the ECM material, making the physical inversion of this elastic distortion an ill-posed problem. However, we find using the following 3D analysis a generic shape for the organism's section close to and in contact with the mounting slide, and propose a simple, general distortion correction mechanism which we use to verify that the conclusions drawn from 2D features are accurate reflections of the 3D-corrected shapes.

E.1. Extraction of volumetric compartment hull from voxel data. The PhII:yfp-stained CZ3 compartment boundaries appear in fluorescence z-stacks as local maxima in intensity, providing a basis from which to extract the bounding shape (convex hull) of the overall compartment geometry by thresholding. This procedure is implemented as follows.

1. Apply masking to z-stacks using the outer boundaries identified in Fig. S5 to isolate the spheroid of interest in 3D space.
2. Equalize intensity across successive z-planes (whose mean generally drops off further in z) by multiplying intensity data of yfp emission at plane z by the ratio μ_z/μ, where μ_z is the mean intensity at plane z and μ is that of the entire volume.
3. Apply a top-hat box transform to subtract background features from the voxel data with radius large compared to the scale of compartent wall thicknesses (on the order of $\sim 2\mu$m as estimated in the main text, p. 4).
4. Obtain an intensity mask by Otsu thresholding (17).
5. Obtain an approximate triangulated surface using the marching cubes algorithm (18).
6. Compute the convex hull of this surface to obtain a convex triangulated surface representing the outer boundary of the CZ3 geometry. Extract the vertices of this hull for use in the following section.

We find consistently that the resulting shape is well-approximated by an ellipsoidal section bounded by a planar slice as shown in Fig. S17; this plane is where the majority of surface compartment features appear and the section in which segmentation (Fig S5, blue dots) is feasible.

Not all spheroids analyzed are sufficiently stably mounted in order to facilitate consistent extraction of the 3D shape from volumetric fluorescence data (nevertheless, surface CZ3 analysis as in the main text is possible from a single or a tight range of focal planes); the ones for which it is feasible are shown in Fig. S5.

E.2. Fitting the ellipsoidal section. We now assume that the organism body is mirror-symmetric about some plane $z > 0$; this z is unknown as it depends on the exact spacing between the two surfaces mounting the spheroid. However, we present here a method for estimating it.

1. For a plane at some height $z > 0$ parallel to the XY plane, make a mirror-image of the vertices extracted in §2.E.1 about it.
2. Compute the minimum-volume bounding ellipsoid containing this point cloud $\{x_i\} \in \mathbb{R}^{n\times 3}$ by solving the convex program

$$\max \log\det A \qquad [38]$$

$$\text{subject to} \; \|Ax_i + b\| \le 1 \;\forall i,\;\; A \ge 0 \qquad [39]$$

   where the resulting ellipsoid (M, v) defined by the equation $(x-v)^\intercal M(x-v) = 1$ is given by the solution as $M = A^\intercal A$, $v = -A^{-1}b$. $A \ge 0$ means that A is positive semidefinite.
3. Compute the cost of the original points not passing through the surface as

$$c = \sum_i ((x_i - v)^\intercal M(x_i - v) - 1)^2 \qquad [40]$$

4. Repeat steps 1-3 with perturbations in the height z to find a fit with locally minimum cost c.

As shown in Fig. S17, this ellipsoidal section is a good approximation of the vertices obtained from the CZ3 convex hull obtained in §2.E.1

E.3. Projection of the compartment geometry. We first note that cells do not experience the same geometric distortion in top view as compartments due to compression because (1) they are approximately spherical objects with similar cross-section from any viewing angle and (2) they are not (significantly) physically deformed by the mounting procedure—rather the outer structures which contain them, i.e. the cellular zone (CZ) and boundary zone (BZ), are. Therefore, the following projections are performed only for the compartment geometry.

1. Given the ellipsoid (M, v) computed in §2.E.2, translate v in z so $v_z > r_{\max}$, where $r_{\max}$ is the maximum of the elliptic radii $1/\sqrt{\lambda_i}$, where $\lambda_i > 0$ are the eigenvalues of M. This ensures (M, v) lies in the upper-half z space.

2. For each polygon in the XY plane defined as a set of points $\{(x_i, y_i)\}_i$, identify the coordinate z_i projected onto the ellipsoid by solving the quadratic equations $w_i^\intercal M w_i = 1$, where $w_i = (x_i, y_i, z_i) - v$. This is solved using Vieta's formula to avoid floating-point cancellation. The roots z_i with smallest value are chosen.

3. The resulting surface $\{(x_i, y_i, z_i)\}_i$ is no longer planar, so find the best-fit plane using the standard SVD solution (12) and take the orthogonal projection of these points onto the plane. This results in a set of CZ3 compartment polygons each lying in their own plane approximately tangential to the ellipsoidal surface computed in §2.E.2. All downstream analyses can then be computed within a basis defined by this plane.

This projection is visualized in Fig. S17.

3. Supplementary analyses

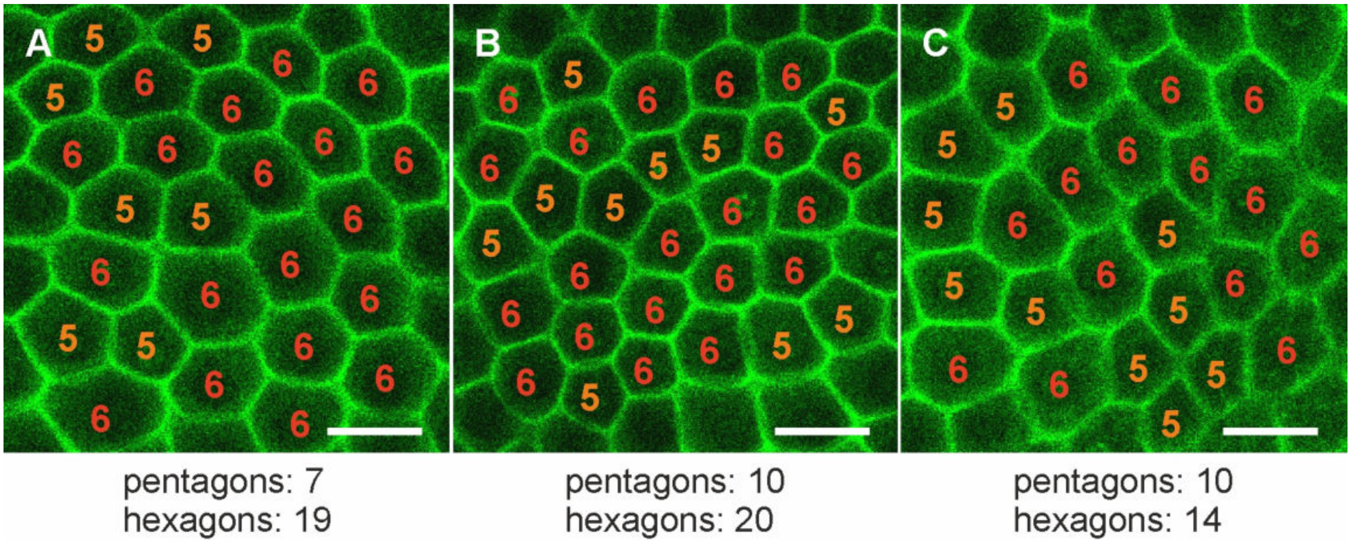

Fig. S4. Share of pentagonal and hexagonal somatic CZ3 compartments in middle aged adults (early stage II). Sexually induced transformants expressing the *phII:yfp* gene under the control of the endogenous *phII* promoter were analyzed in vivo for the localization of the PHII:YFP fusion protein. Magnified view of the PhII:YFP-stained compartments surrounding the somatic cells corresponding to CZ3. In areas where no gonidia lie below the somatic cell sheet, the compartments form a pattern of hexagons and pentagons. In the exemplary regions shown here, 27 pentagonal and 53 hexagonal compartments were counted, representing a ratio of roughly 1:2. Scale bars are 20 μm.

	**Spheroid**						
**Stage I**	I.1	I.2	I.3	I.4	I.5	I.6	I.7
Circular radius (μm)	96	107	104	109	103	117	108
Segmented cell-compartment pairs	152	317	267	260	265	249	248
**Stage II**	II.1	II.2	II.3	II.4	II.5		
Circular radius (μm)	188	230	221	247	219		
Segmented cell-compartment pairs	397	494	548	535	495		
**Stage III**	III.1	III.2	III.3	III.4	III.5		
Circular radius (μm)	245	243	266	241	223		
Segmented cell-compartment pairs	447	404	520	439	437		
**Stage IV**	IV.1	IV.2	IV.3	IV.4	IV.5		
Circular radius (μm)	416	419	419	428	430		
Segmented cell-compartment pairs	519	446	430	550	529		
**Stage S**	S.1	S.2	S.3	S.4	S.5	S.6	S.7
Circular radius (μm)	254	281	269	278	205	287	280
Segmented cell-compartment pairs	415	466	497	531	396	580	551

Table S1. Summary of individual spheroid data: equivalent circular radius and number of analyzed cell-compartment pairs.

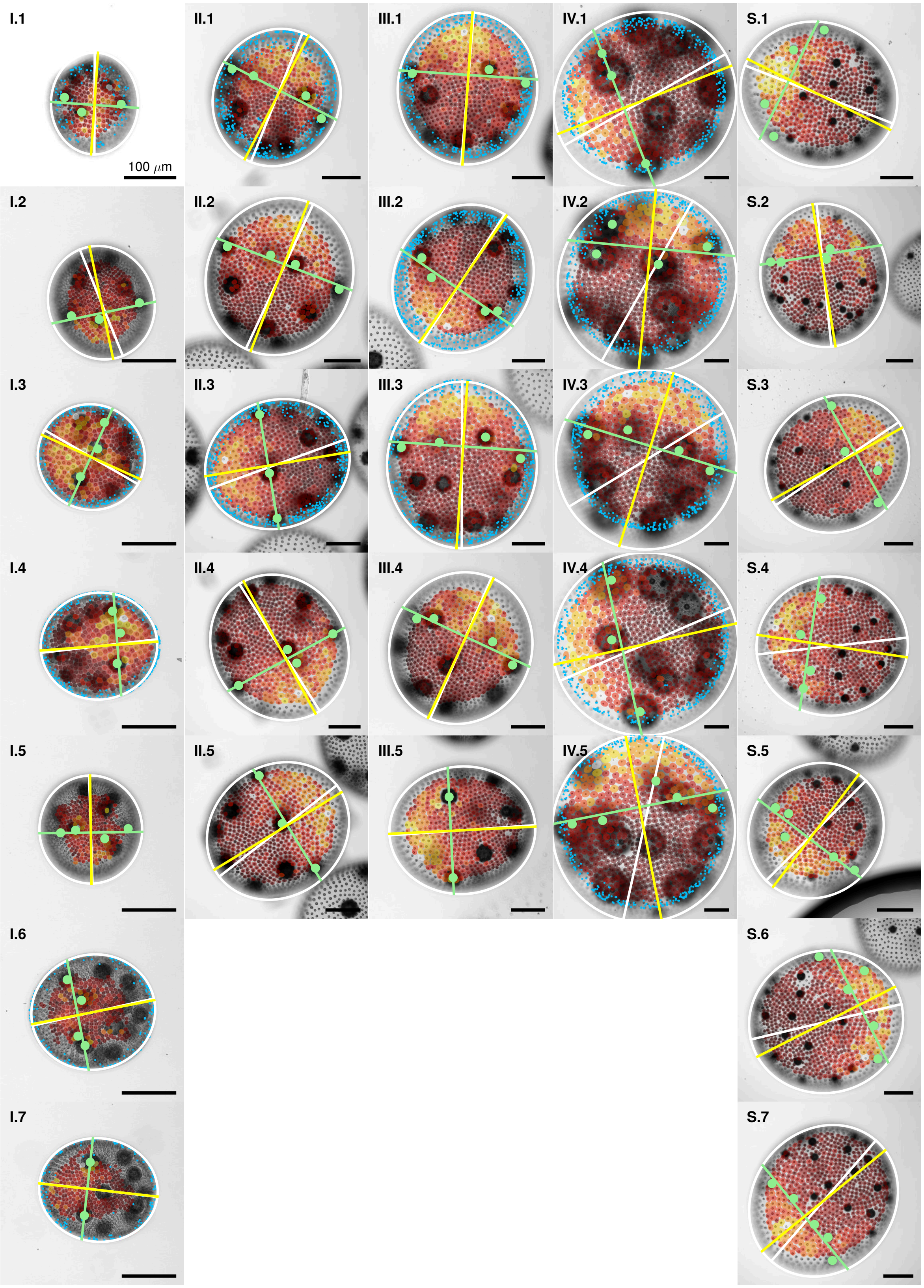

Fig. S5. Segmented PhII:YFP signal and anterior-posterior axis identification. Trans-PMT images of spheroid in stage I-IV and S (defined in Fig. 3 in the main document) with posterior-anterior axis (yellow line) estimated as the line passing through the elliptical (white outline) center which is normal to the best-fit line (green) through manually identified offspring (green dots) located in the anterior section. The white line represents the major axis of the ellipsoid. Overlaid are segmentations of the CZ3 compartments (PhII:YFP), colored by area (dark to light by size). Blue dots are the (x, y) coordinates of the CZ3 convex hull coordinates as defined in §2.E.2 (in the spheroids from which extraction is possible) and whose full 3D coordinates are also rendered in Fig. S17. The approximately elliptical middle regions where points are absent are the flat contact patches between organisms and cover slips (Materials and Methods, main text), defining the feasible segmentation region.

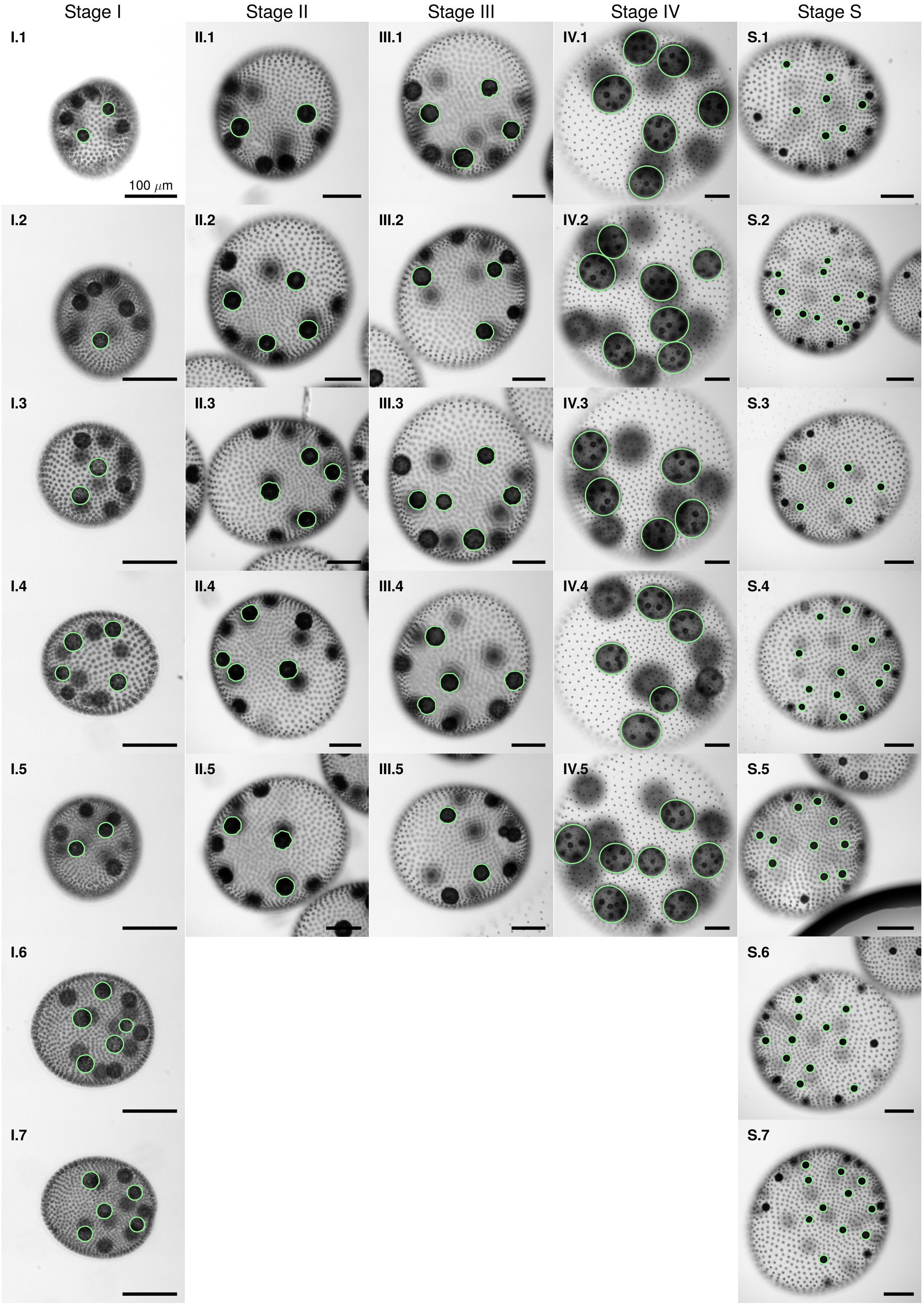

Fig. S6. Offspring. Offspring are identified using the same semi-automated procedure as that used for identification of somatic cells and CZ3 compartment geometry. Being far larger than either of those, however, offspring appear in focus at different planes. We identify offspring from planes where clearly in focus, displaying here particular planes for each spheroid. The boundaries are used to estimate the volumetric growth rates displayed in Table 2, main text.

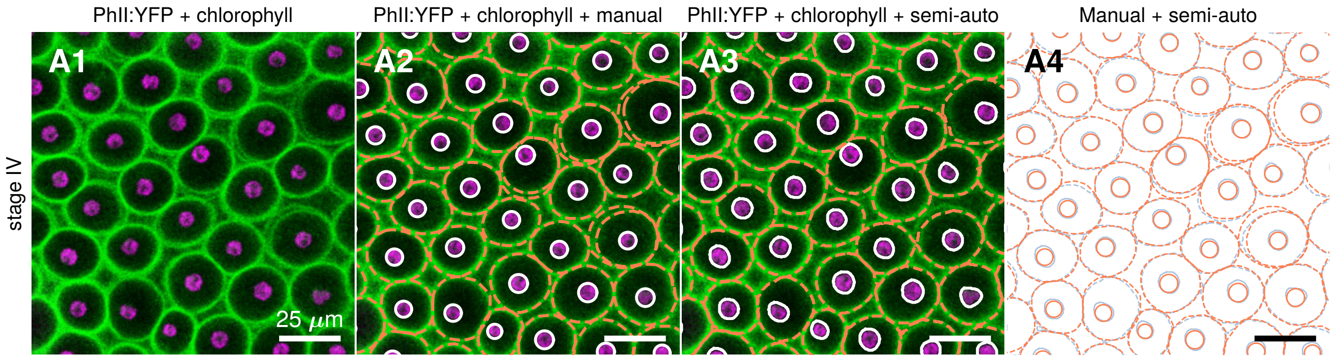

Fig. S7. Comparison of semi-automated segmentation against fully manual segmentation. 1. Overlay of YFP fluorescence of PhII:YFP protein (green) and chlorophyll fluorescence (magenta), detected at 650-700 nm. 2. Same as 1 with *manually* segmented CZ3 (orange) and cell boundaries (white), identical to panel A2, Fig. 5 in the main text. 3. Same as 2 with *semi-automatically* segmented (using the procedure described in §2) CZ3 (orange) and cell boundaries (white). 4. Comparison of semi-automated segmentation (blue, CZ3 compartments and cells) with fully manual segmentation (orange, CZ3 compartments and cells).

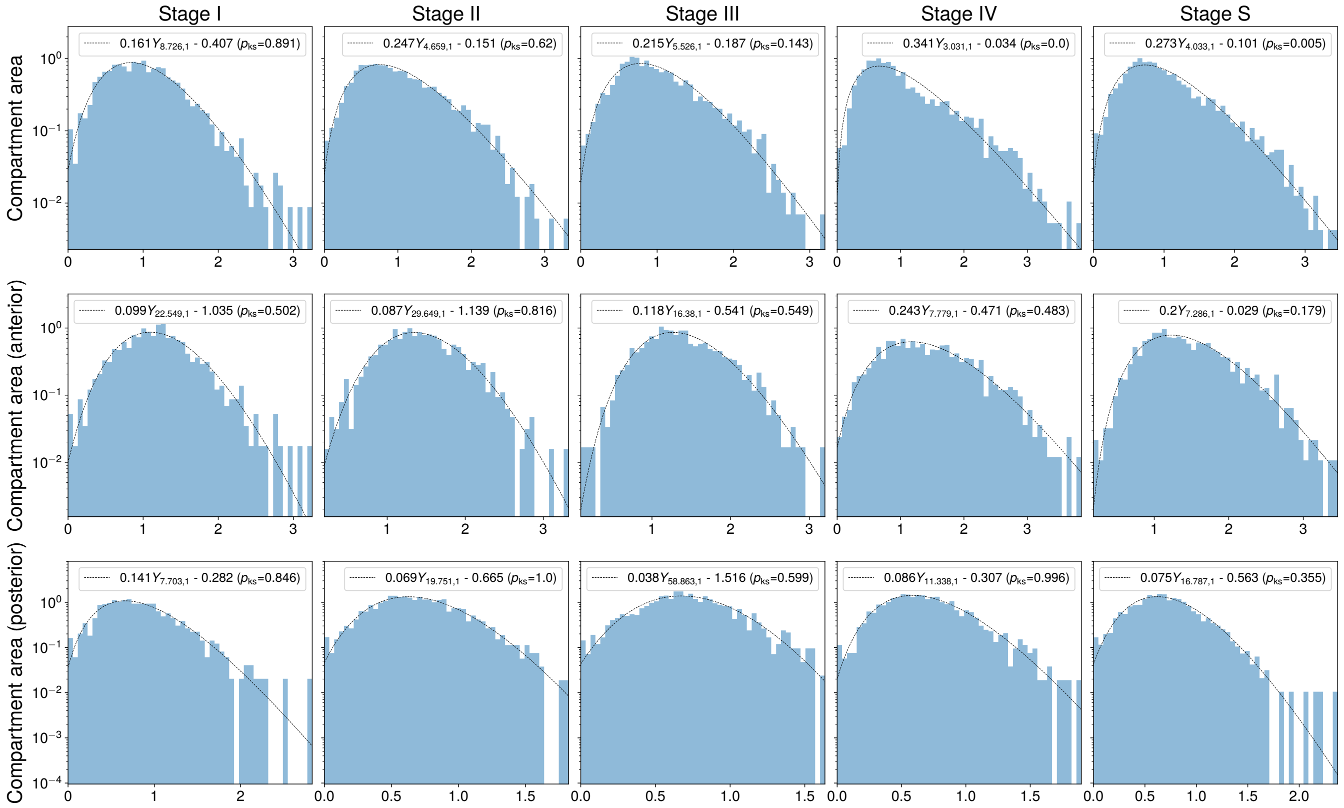

Fig. S8. Areas are gamma-distributed throughout growth with changing shape parameters reflecting anterior-posterior differentiation. Areas of CZ3 compartments robustly follow gamma-distributions, as highlighted in Fig. 8B2 and §E, main text. Plotted on y-axes (shared by row) are normalized counts per bin ($n = 50$ bins), and on x-axes are the reduced CZ3 areas $\tilde{a}_{\mathrm{cz3}} = (a_{\mathrm{cz3}} - a_{\mathrm{min}})/(a_{\mathrm{avg}} - a_{\mathrm{min}})$, where a_{min} and a_{avg} are minimum and average values of a_{cz3} for the respective spheroid. Such normalization enables distributional fit across different organisms. The empirical mean is therefore fixed at 1 in these distributions, and the maximum-likelihood fit parameters are k and offset of the support (with λ determined by the relation between k and the fixed mean). A Kolmogorov-Smirnov (KS) goodness-of-fit test is performed and its p-value is recorded as p_{ks} in each plot. One key observation we make, evident in the first row, is that the extreme degree of anterior-posterior differentiation (shown in Fig. 8B1, main text) suggests that a single fit from this distribution family may not be valid in later stages of the life cycle. Indeed, $p_{\mathrm{ks}} \approx 0.9$ in stage I, indicating no strong evidence that $\tilde{a}_{\mathrm{cz3}}$ do not arise from a gamma distribution, yet drops below 0.001 by stage IV, supporting rejection of the fit hypothesis in this case. Qualitatively, one observes the formation of "shoulders" in the distribution (exhibiting a loss of log-concavity which does not hold in the gamma distribution for shape parameter $k \geq 1$). Separating the data by anterior/posterior hemispheres, however, almost completely resolves the issue and reveals that the respective $\tilde{a}_{\mathrm{cz3}}$ values follow gamma distributions (of very different shape parameters). Lastly, fitted parameters of the sexual stage S place it distributionally closer to Stage IV than to III, which contrasts with the grouping inferred from the spheroid radii (Fig. 10E, main text).

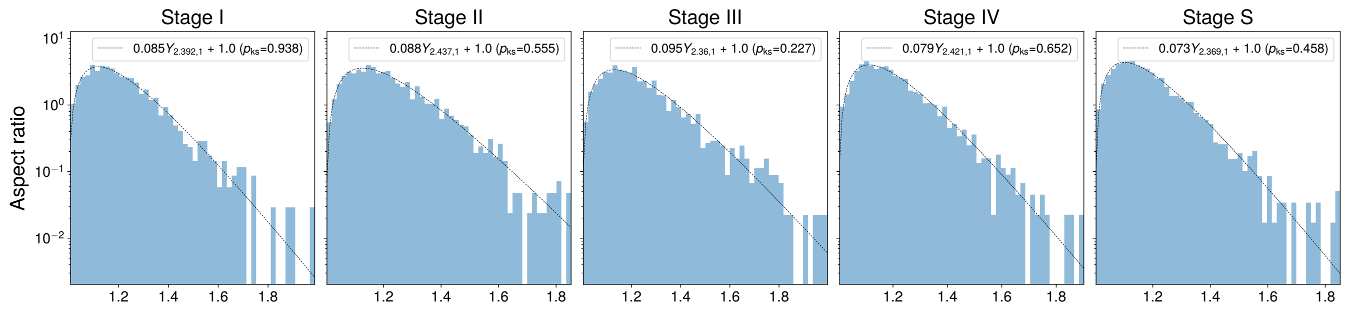

Fig. S9. Aspect ratios remain stably gamma-distributed throughout growth. As seen, the k parameter remains in a remarkably tight range between $2.35 - 2.45$. Maximum-likelihood estimated (MLE) parameters include only rate λ and shape k, with location fixed at 1. Both this distribution family and the value of the shape parameter for aspect ratios are robustly observed in a variety of living organisms and inert jammed systems (19). Kolmogorov-Smirnov tests performed in each case indicate no strong evidence that the data do not follow gamma distributions.

Stage	Spheroid volume change (est., mm^3)	Total offspring volume change (est., mm^3)	Total cell volume change (est., μm^3)	% spheroid volume change to offspring (est., %)	% spheroid volume change to cells (est., %)	Elapsed time (h)	Spheroid growth rate (est., mm^3/h)	Total offspring volume growth rate (est., mm^3/h)	Total cell volume growth rate (est., μm^3/h)
I	$\downarrow$	$\downarrow$	$\downarrow$	$\downarrow$	$\downarrow$	$\downarrow$	$\downarrow$	$\downarrow$	$\downarrow$
II	0.0403	0.0011	3.1×10^5	2.8	0.76	15	0.0027	7.5×10^{-5}	10.2
III	0.0153	0.0002	4.2×10^4	1.2	0.28	6	0.0025	3.1×10^{-5}	3.5
IV	0.2553	0.0258	7.9×10^4	10.1	0.03	16	0.0160	1.6×10^{-3}	2.5

Table S2. Estimated volumetric growth changes by life cycle stage (I-IV), supplementary to Table 2, main text. Number of juveniles per spheroid were manually estimated from the trans-PMT image (as in Fig. S6), with the average value over all $n = 29$ spheroids being exactly 13. The estimated total offspring volume change (column 3) is defined as this number, times the average estimated juvenile volume per stage (as given in Table 2, main text, computed from segmentation of the trans-PMT image, Fig. S6). Further, assuming a fixed count of $2^{11} \approx 2000$ somatic cells per spheroid and multiplying this times the average somatic cell volume per stage (again Table 2, main text) yields the estimated total volume change due to somatic cells (column 4). Assuming 2^{12} somatic cells changes the contribution to the overall spheroid volume negligibly, as evident from column 6. Taken together, columns 2, 3, and 4 yield an estimation of the contribution to overall spheroid volume change by offspring, somatic cells, and parental ECM (Table 2, column 5, main text) by subtracting columns 3 and 4 from 2. Lastly, knowledge of the approximate elapsed time between life cycle stages (column 7) yield the corresponding estimated growth rates (columns 8-10 here and Table 2, columns 5-6, main text).

	Cell area	Compartment area	Aspect ratio	Circularity	Offset	Offset (whitened)	Voronoi error
% change PA in I	13.72	44.25	9.85	-2.49	46.90	22.59	1.66
% change PA in II	13.44	93.90	3.06	-0.69	117.00	65.15	15.61
% change PA in III	13.28	121.88	1.01	-0.67	82.04	36.93	-6.55
% change PA in IV	8.12	129.56	14.39	-0.53	49.86	8.72	-10.31
% change PA in S	9.90	128.90	-1.76	-1.94	195.36	105.17	-3.06
Mean value in stage I	26.37	57.85	1.20	0.88	0.54	0.24	0.21
% change mean I to II	81.47	201.09	0.93	1.95	141.60	43.29	21.60
Mean value in stage II	47.85	174.18	1.21	0.90	1.29	0.35	0.25
% change mean II to III	2.38	30.02	0.86	-0.78	27.36	12.05	-1.41
Mean value in stage III	48.99	226.48	1.22	0.89	1.65	0.39	0.25
% change mean III to IV	12.21	165.02	-2.74	1.82	52.87	-5.58	15.60
Mean value in stage IV	54.97	600.21	1.19	0.91	2.52	0.37	0.29

Table S3. Summary of changes in mean value along posterior-anterior axis (PA) and by life cycle stage (I-IV, S). Rows 1-5 show the changes in empirical mean value from posterior to anterior end (corresponding to dashed mean lines, column 1, Fig. 8, main text) within each life cycle stage . Rows 6-12 show the changes in mean value (corresponding to vertical black bars overlaying the histograms, column 2, Fig. 8, main text) across successive life cycle stages. While the underlying distributions are highly skewed and mean-based comparisons should be interpreted carefully as noted in §E1 (main text), they provide one quantification of the underlying trends visible in the empirical distributions, Fig. 8, main text.

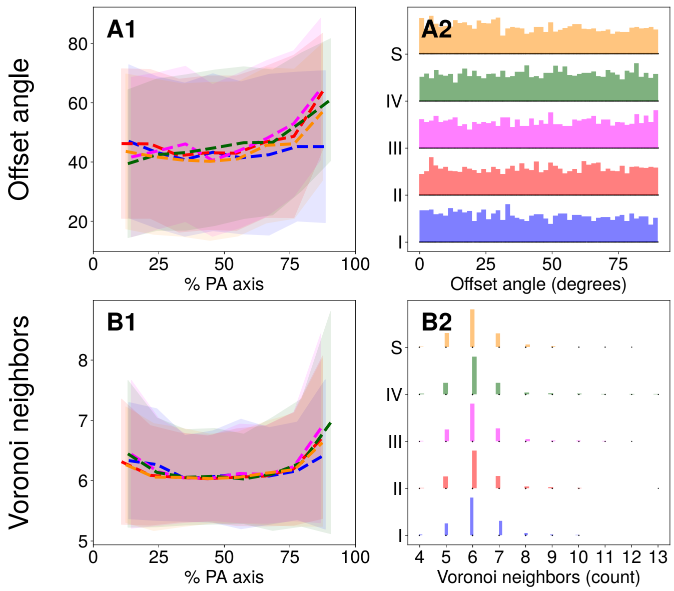

Fig. S10. Additional invariances in the stochastic geometry of the CZ3 compartments across the life cycle. Rows A and B follow the same formatting as Fig. 8, main text. Row A shows variation in the offset angle $\theta_{\mathrm{cell}} = \arccos(|\Delta\boldsymbol{x} \cdot \boldsymbol{v}| / (\|\Delta\boldsymbol{x}\| \, \|\boldsymbol{v}\|))$ between the somatic cell offset vector $\Delta\boldsymbol{x}$ (Table 1, main text) and the principal stretch axis $\boldsymbol{v}$ of the CZ3 compartment (given by the eigenvector corresponding to $\lambda_{\max}$). Since the latter has no polarity, $\theta_{\mathrm{cell}} \in [0, 90]$ degrees. Panel A1 shows that there is little variation along the PA axis or by life cycle, with the exception of upward tails toward the anterior pole in stages II-IV and S. Panel A2 reveals furthermore that θ_{cell} effectively follows a uniform distribution over its support throughout the lifcycle, indicating that there is no correlation between the somatic cell offset vector $\Delta\boldsymbol{x}$ and the principal stretch axis. This is perhaps surprising given that one might naively expect translation of the cell along the deformation axis of its compartment. Row B shows, in the same format, the number of neighbors (edges) of each Voronoi partition. Panel B1 again shows that there is almost no variation along the PA axis or by life cycle stage, providing quantitative evidence that the topology of the CZ3 space partition does not change during growth. Panel B2 displays the distribution of Voronoi neighbors by life cycle stage, whose mean is around 6, consistent with Euler's theorem for Voronoi tessellations of the sphere (§D).

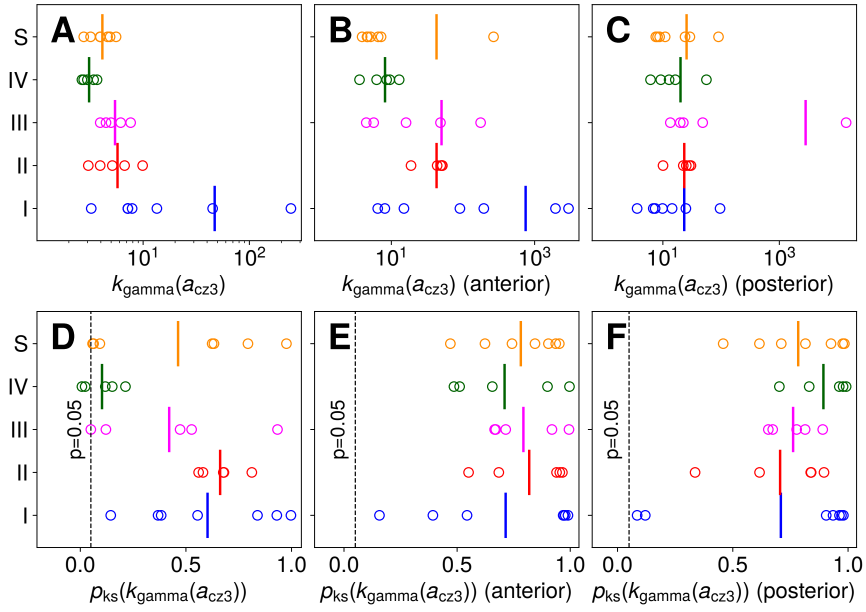

Fig. S11. Increasing area polydispersity during the life cycle is primarily due to changes in the anterior hemisphere. Panel A is the same as Fig. 10B (main text) while panels B and C show the same metrics separated by anterior and posterior regions respectively. As seen, B shows a decrease in k (in increase in disorderedness as explained in main text) of the anterior configuration by life cycle stage, while C shows relatively stable values in the posterior configuration. The former therefore likely underlies the increasing disorderedness observed in the whole spheroid (panel A). Panels D-F display the respective p-values (p_{ks}) of a Kolmogorov-Smirnov goodness-of-fit test performed in each instance of the k_{gamma} value reported in panels A-C. All panels D-F show that stage I can exhibit varying degrees of goodness-of-fit, which is likely reflected by the large spread of k-values in stage I (panels A-C). However, in the progression from stages II-IV and S, p_{ks} drops significantly, sometimes below the threshold (plotted as dashed vertical lines), as expected from Fig. S8, despite the qualitatively good match observed in row 1 of that figure. As in Fig. S8 rows 2-3, this is remedied by separating into anterior/posterior hemispheres, at which point the data collapses well, indicated by the sharp increases in p_{ks} in panels E-F. Remarkably, this increase in goodness-of-fit accompanies a clear observation in panels B-C that the anterior hemisphere is primarily responsible for increasing disorder in the somatic CZ3 configuration.

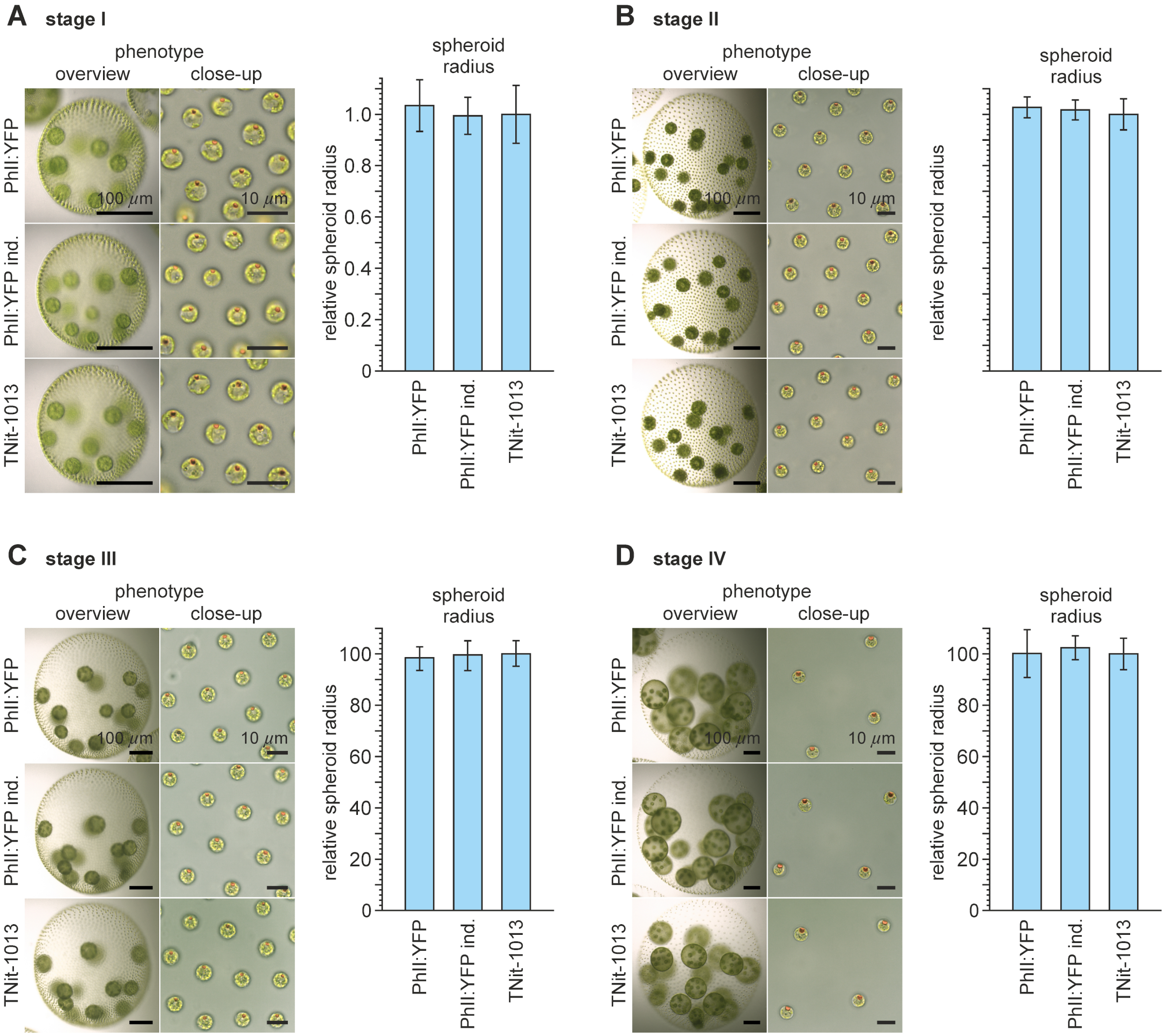

Fig. S12. Phenotypes of PhII:YFP-expressing transformant and recipient (control) *V. carteri* strains at developmental stages I to IV. The phenotypes of transformant PhII:YFP and the recipient TNit-1013 (control) strains were compared to check whether the expression of the PhII:YFP fusion protein alters the phenotype. It was also investigated, whether the addition of the sex-inducer protein for only 24 h (inducer added, ind.), i.e. for the period used in the experiments, already leads to a change in the vegetative phenotype of PhII:YFP transformants. (A-D) Developmental stages I to IV. Representative light microscopic images on the left show overviews and close-ups. The overviews reveal the spheroid morphology, spheroid size, expansion kinetics, the arrangement of the progeny and the regularity of the arrangement of the somatic cells. The close-ups show the regularity of the arrangement of the somatic cells in detail, the size of the somatic cells and the orientation of the eyespots. Bar charts on the right show the analysis of the spheroid radii. The average spheroid radius is shown in relation to the recipient (control) strain TNit-1013 (=1.0). The standard deviation is indicated (n=20). There is no evidence of changes in phenotype due to the expression of PhII-YFP or the addition of the sex-inducer protein for 24 h.

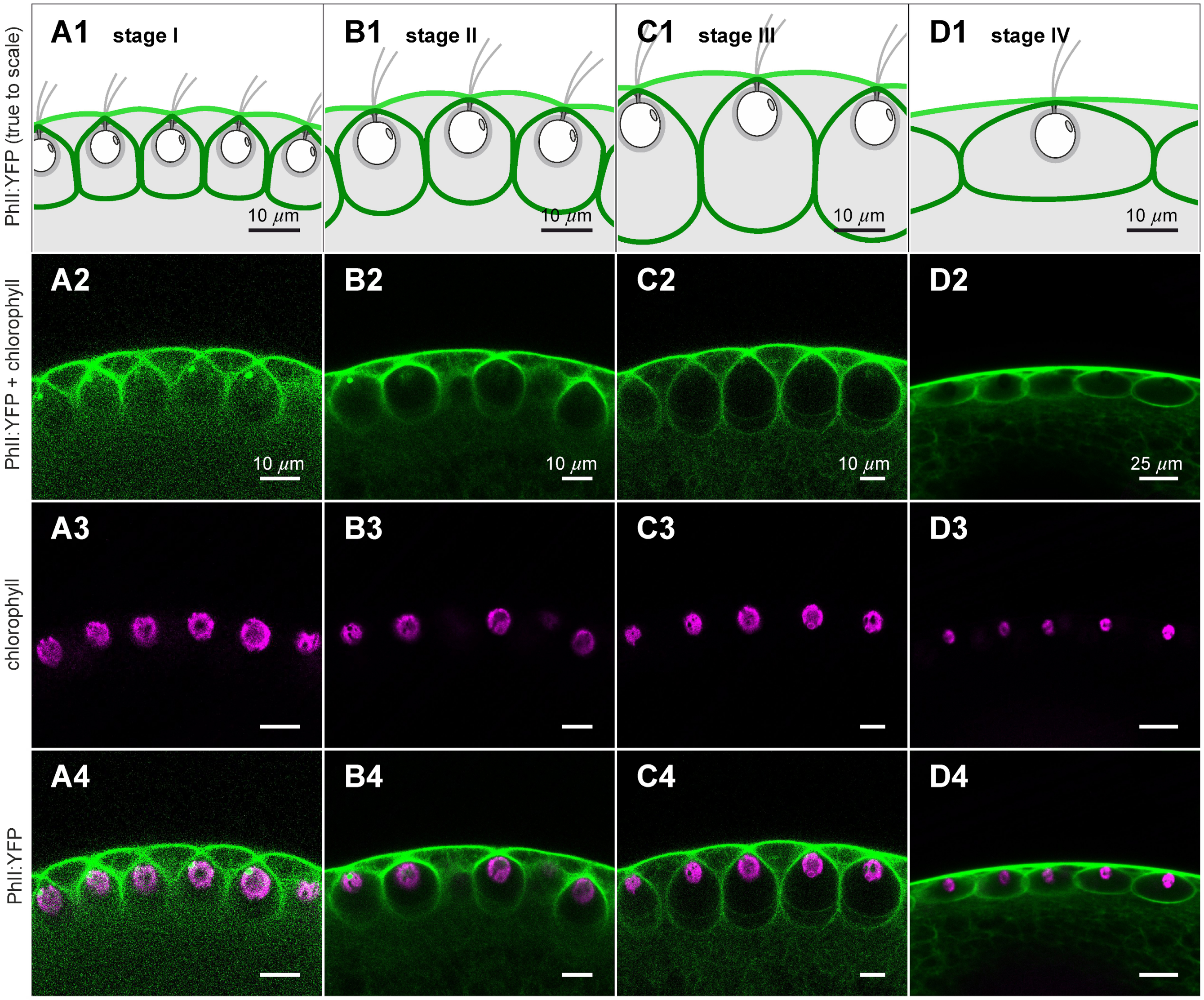

Fig. S13. Cross sections through PhII:YFP-stained somatic CZ3 compartments at developmental stages I to IV. Sexually induced transformants expressing the *phII:yfp* gene under the control of the endogenous *phII* promoter were analyzed in vivo in developmental stages I-IV (columns 1-4) for the localization of the PHII:YFP fusion protein. Side view orthogonal to the geometrically analyzed top view showing the shapes of the CZ3 compartments along the Z-axis of the top view. PhII:YFP is located in the CZ3 of somatic cells and in the BZ. It should be noted that when looking at all cLSM images taken as a whole, a considerable proportion of the "floors" of the chambers are poorly recognizable or appear as a diffuse, unclear boundary. Row 1 (A1-D1): True-to-scale schematic cross section, showing PhII:YFP localization in CZ3 of somatic cells (dark green) and BZ (light green). The somatic cells and further ECM material are shown in white or gray. Row 2 (A2-D2): YFP fluorescence of the PhII:YFP protein (green). Row 3 (A3-D3): Chlorophyll fluorescence (magenta). Row 4 (A4-D4): Overlay of YFP and chlorophyll fluorescence.

Stage	Time (h)	Cross-sectional major axis length (μm)	Cross-sectional minor axis length (μm)	Cross-sectional area (μm^2)	Cross-sectional aspect ratio	Cross-sectional elongation	Ellipsoidal est. volume (μm^3)
I	0	14.06	11.82	130.90	1.20	Radial	1045.19
II	15	19.45	17.57	268.30	1.11	Radial	3145.50
III	21	30.20	23.50	557.51	1.29	Radial	8750.71
IV	37	38.91	18.75	572.54	0.48	Tangential	14892.58

Table S4. Estimates of cross-sectional shape from developmental stages I to IV. As seen from Fig. S14 columns B-C, compartment cross sections can form double-walls. We use the outer wall in these estimates, which also appears of generally higher fluorescent intensity. Compartment cross-sections are segmented (where feasible in the sections shown in Fig. S14) into polygons whose major and minor axis lengths are computed from the second area moment as in §2.B.2, reported in columns 3 and 4. Columns 3-8 are averages across the polygons within each image. Column 6 indicates the aspect ratio defined as the ratio of radial and tangential axes. Column 7 indicates whether the compartment's major axis is oriented radially or tangentially with respect to the spheroid. Column 8 is an estimate of the compartment's 3D volume as an ellipsoid with cross-sectional axes as in columns 3 and 4, revolved around the radial axis.

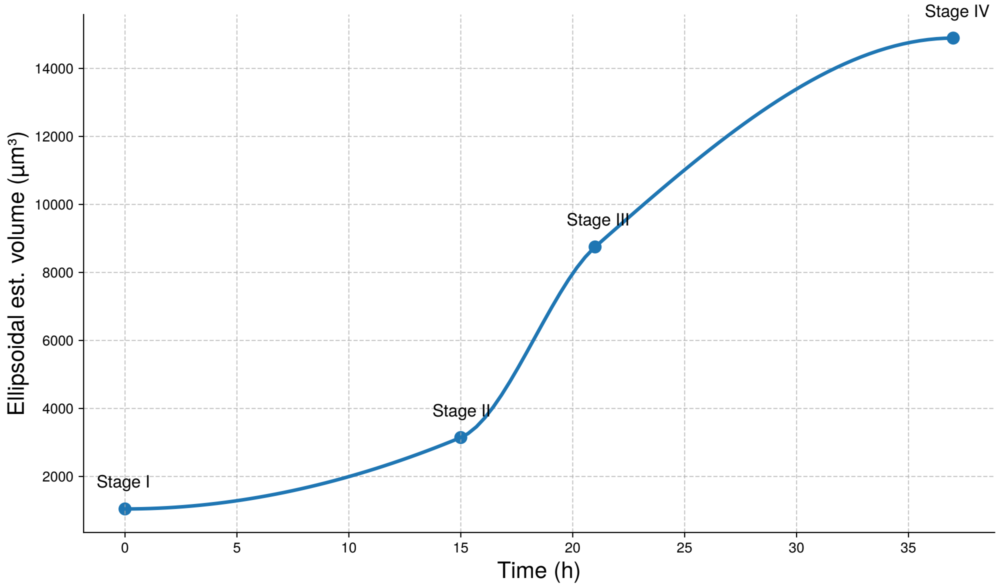

Fig. S14. Interpolated volumetric growth curve estimated from cross-section. Columns 2 (time) and 8 (estimated compartment volume) from Table S4 are shown with a monotone interpolant (piecewise cubic Hermite polynomial). As seen, estimated volumetric growth accelerates between Stages II and III, displaying an overall sigmoid-like shape.

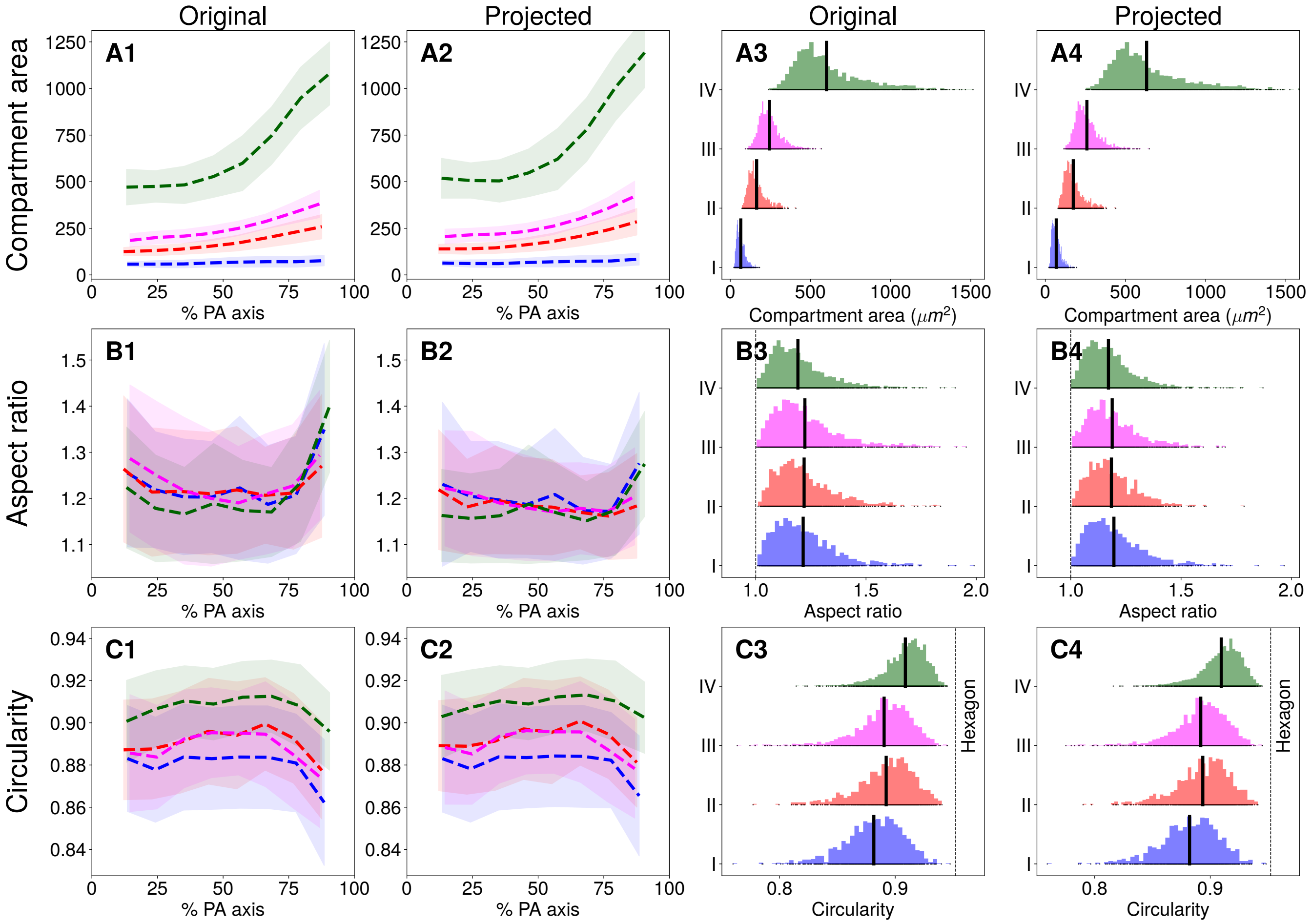

Fig. S15. Comparison of original and projected compartment metrics. From the subset of spheroids in which accurate extraction of the 3D geometry is possible as described in §2.E.1 (in particular none from stage S), the distortion correction procedure described in §2.E.3 is applied to the compartment polygons and plots identical to those in Fig. 8, main text, are shown for these polygons. X- and Y-axes are matched between "Original" and "Projected" columns. As seen, the only noticeable variation between the original and projected compartment features are in the extreme anterior region, particularly in stage IV, with respect to area and aspect ratio. Area (A1-2) slightly increases in the projected compartments compared to the original. Aspect ratio (B1-2), which showed upward spikes at the anterior and posterior extremes in the original geometry (B1) now shows a more flat response throughout the AP axis in the projected geometry (B2), as expected. Other than a loss of right outliers in aspect ratio (B3-4) and left outliers in circularity (C3-4) the distributions of these features (columns 3-4) is essentially unchanged between the original and projected geometries. In particular, the aspect ratio distributions are even more stable in mean in the projected geometry relative to the original geometry, from which we first made this observation (main text).

Stage	Compartment area (original) (μm^2)	Compartment area (projected) (μm^2)	Aspect ratio (original)	Aspect ratio (projected)	Circularity (original)	Circularity (projected)
I	65.69	68.53	1.22	1.20	0.88	0.88
II	164.56	174.71	1.22	1.18	0.89	0.89
III	244.12	259.56	1.22	1.19	0.89	0.89
IV	600.21	632.07	1.19	1.17	0.91	0.91

Table S5. Quantitative comparison of original and projected compartment metrics by mean. Reflecting the black bars showing empirical means in Fig. S15, we find minimal change in aspect ratio and circularity between original and projected geometries. Compartment area shows consistently around a 5% increase from the original to the projected geometry.

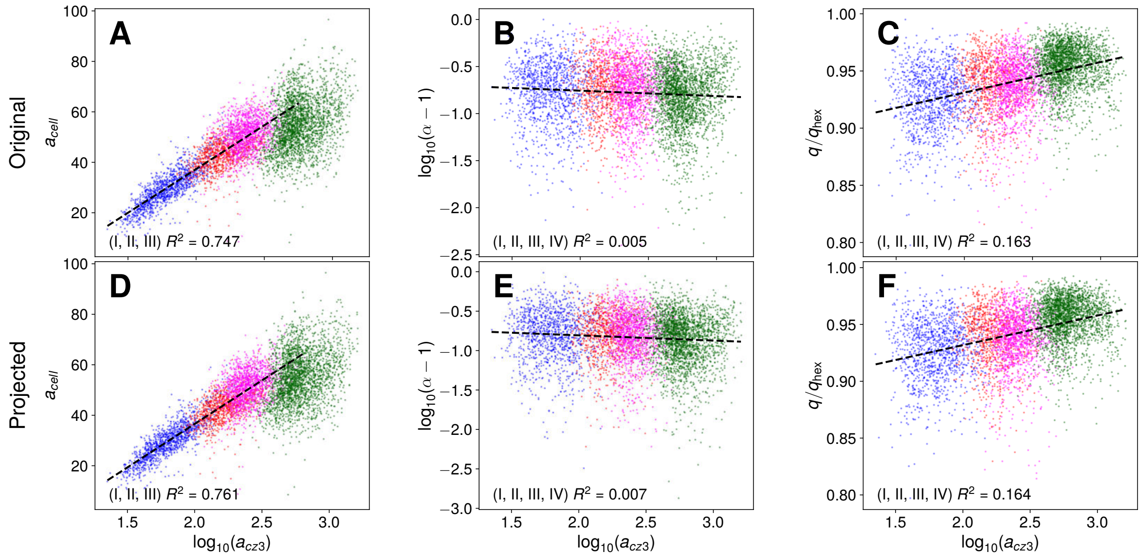

Fig. S16. Comparison of feature correlations in original and projected compartment geometries. Correlations between compartment area (a_{cz3}) and various metrics (cell area (a_{cell}), aspect ratio (α), and circularity (q) in column order) are shown, as in the main text Fig. 9. As shown, minimal change is present from the original to the projected compartment geometries, with a slight increase in correlation between cell and compartment area in the projected geometry (D), which is consistent with the slight increase in compartment area as seen in Fig. S15 and Table S5. As discussed in §2.E.3, projection is performed only for compartment geometry, as cellular cross-sections are not deformed by the mounting procedure; a_{cell} is identical in panels A and D.

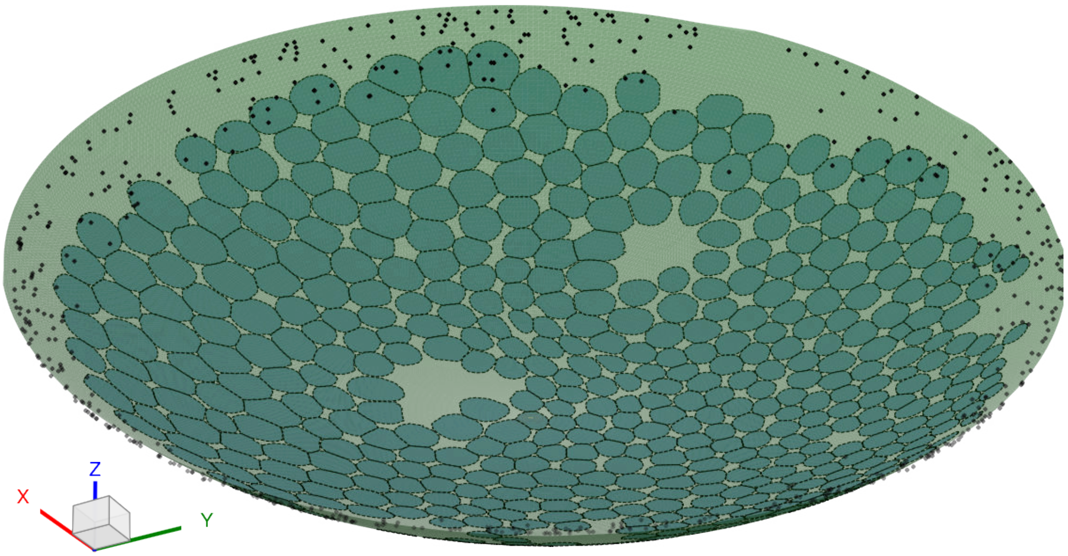

Fig. S17. Elastic distortion correction by 3D projection of surface features onto ellipsoidal cap. Black points are the vertices of the yfp 3D convex hull defined in §E.1. The green shell is the ellipsoidal cap defined in §E.2. Blue regions are the compartment geometry projected onto this surface as defined in §E.3. As seen, the inferred ellipsoidal sections are in good agreement with hull points extracted from 3D fluorescence intensity data. The region in the middle in which few hull points appear is the planar section of compression of the organism against the glass slide, as seen from top view in Fig. S5. The ellipsoidal cap (green) "re-inflates" this region. The shown data is from a spheroid in stage IV, in which we expect the greatest amount of distortion due to the organism's size.

References

1. B von der Heyde, A Hallmann, Targeted migration of pherophorin-S indicates extensive extracellular matrix dynamics in *Volvox carteri*. *The Plant J.* **103**, 2301–2317 (2020).
2. SE Prochnik, et al., Genomic analysis of organismal complexity in the multicellular green alga *Volvox carteri*. *Science* **329**, 223–226 (2010).
3. DM Goodstein, et al., Phytozome: a comparative platform for green plant genomics. *Nucleic acids research* **40**, D1178–D1186 (2012).

4. B Klein, D Wibberg, A Hallmann, Whole transcriptome RNA-Seq analysis reveals extensive cell type-specific compartmentalization in *Volvox carteri. BMC Biol.* **15**, 111 (2017).
5. A Hallmann, Extracellular matrix and sex-inducing pheromone in *Volvox. Int. Rev. Cytol.* **227**, 131–182 (2003).
6. S El-Gebali, et al., The pfam protein families database in 2019. *Nucleic acids research* **47**, D427–D432 (2019).
7. SC Potter, et al., Hmmer web server: 2018 update. *Nucleic acids research* **46**, W200–W204 (2018).
8. KJ Lauersen, O Kruse, JH Mussgnug, Targeted expression of nuclear transgenes in *Chlamydomonas reinhardtii* with a versatile, modular vector toolkit. *Appl. Microbiol. Biotechnol.* **99**, 3491–3503 (2015).
9. C Stringer, T Wang, M Michaelos, M Pachitariu, Cellpose: a generalist algorithm for cellular segmentation. *Nat. Methods* **18**, 100–106 (2021).
10. C Stringer, M Pachitariu, Cellpose3: one-click image restoration for improved cellular segmentation. *bioRxiv* (2024) Preprint.
11. P Milanfar, GC Verghese, WC Karl, AS Willsky, Reconstructing polygons from moments with connections to array processing. *IEEE Transactions on Signal Process.* **43**, 432–443 (1995).
12. G Strang, *Introduction to Linear Algebra, Sixth Edition.* (Wellesley-Cambridge Press, Philadelphia, PA), (2022).
13. N Fusco, F Maggi, A Pratelli, The sharp quantitative isoperimetric inequality. *Annals Math.* pp. 941–980 (2008).
14. G Polya, G Szegö, *Isoperimetric Inequalities in Mathematical Physics.* (Princeton University Press, Princeton), (1951).
15. L Fejes Toth, Sur la représentation d'une population infinie par un nombre fini d'éléments. *Acta Math. Acad. Sci. Hungarica* **10**, 299–304 (1959).
16. D Newman, The hexagon theorem. *IEEE Transactions on Inf. Theory* **28**, 137–139 (1982).
17. N Otsu, , et al., A threshold selection method from gray-level histograms. *Automatica* **11**, 23–27 (1975).
18. WE Lorensen, HE Cline, Marching cubes: a high resolution 3D surface construction algorithm in *Seminal graphics: pioneering efforts that shaped the field, Volume 1.* (Association for Computing Machinery, New York, NY, USA) Vol. Volume 1, pp. 347–353 (1998).
19. L Atia, et al., Geometric constraints during epithelial jamming. *Nat. Phys.* **14**, 613–620 (2018).
